# Meat taxes in Europe can be designed to avoid overburdening low-income consumers

**DOI:** 10.1038/s43016-023-00849-z

**Published:** 2023-10-02

**Authors:** D. Klenert, F. Funke, M. Cai

**Affiliations:** 1 Joint Research Centre of the European Commission, Seville, Spain; 2 Faculty of Economics and Management, Technical University of Berlin, Berlin, Germany; 3 Potsdam Institute for Climate Impact Research, Potsdam, Germany; 4 Institute for New Economic Thinking and School of Geography and the Environment, University of Oxford, Oxford, England

**Keywords:** Economics, Environmental economics, Environmental studies

## Abstract

Consumption taxes on meat have recently been under consideration in several European countries as part of their effort to achieve more sustainable food systems. Yet a major concern is that these taxes might burden low-income households disproportionately. Here we compare different meat tax designs and revenue recycling schemes in terms of their distributional impacts in a large sample of European countries. We find that across all selected tax designs, uncompensated meat taxes are slightly regressive. However, the effect on inequality is mild and can be reversed through revenue recycling via uniform lump-sum transfers in most cases. Using meat tax revenues towards lowering value-added taxes on fruit and vegetable products dampens but does not fully offset the regressive effect. Variation in the distributional impact can be explained by cross-country heterogeneity in consumption patterns, design choices between unit-based and ad valorem taxation and differentiation according to greenhouse gas intensities.

## Abstract (web summary)

Taxes are increasingly used to disincentivize the consumption of specific food products associated with negative impacts for health and the environment. This study compares the distributional effects of different tax designs and revenue recycling mechanisms for taxing meat products across the European Union and United Kingdom.

## Main

Stringent environmental regulation of livestock farming and meat products is notably lacking, despite their contribution to climate change, biodiversity loss, deforestation and nitrogen pollution^1^. Recent assessments suggest that the 1.5 °C climate target set out in the Paris Agreement cannot be attained without rapid and ambitious changes to global food systems^2^. Filling this regulatory gap is increasingly important, especially in high-income regions such as the European Union, where per capita meat consumption is currently at unsustainably high levels^3^.

Against this backdrop, the topic of meat taxation has gained growing political attention in recent years, with the European Commission’s Farm-to-Fork Strategy detailing a vision of an EU tax system that adequately reflects the ‘real cost’ of environmental damages associated with food items. Indeed, the dietary transition necessary to align food systems with environmental objectives will, among other measures, probably require a price signal^4^. In the absence of a comprehensive upstream price on greenhouse gas (GHG) emissions and other agricultural externalities, consumption taxes on meat and other animal products, which are substantially more carbon intensive than other foods^5,6^, are a simple second-best policy option^7^.

However, a recurring argument against taxation of food items is its potential regressivity. As low-income households spend a larger share of their income on food, they are disproportionately affected by food taxes. For precisely this reason, value-added taxes on food and other essential items have been set at reduced rates in many European countries. This begs the question whether an environmental price signal on high-polluting food items will inevitably come at the expense of higher inequality. A disproportionate burden on low-income households threatens the distributional fairness and political feasibility of introducing meat taxes^8,9^, especially in light of rising food prices and inflation, and may exacerbate existing income-related food insecurities, especially in eastern and southern Europe^10^.

Here we investigate these distributional effects. When designing consumption taxes on meat, policymakers are faced with three essential design choices. First, should taxes be ad valorem or based on product units? Second, to what extent can tax rates be differentiated according to environmental impact? Third, what is to be done with the revenues from meat taxation? Our analysis simulates how these choices affect the distributional outcomes from taxing meat consumption using microdata from consumer expenditure surveys in 25 European countries (European Union and United Kingdom).

Whereas a substantive body of research has demonstrated the regressivity of carbon-equivalent food taxes, these studies have either focused on single countries or considered a single tax design^11–15^. By contrast, we compare the relative distributional effects of ad valorem taxes, unit taxes and emissions-based taxes on meat and of different revenue recycling mechanisms in a multi-country context, using household-level expenditure data from the EU Household Budget Survey (EU-HBS). In each scenario, we compare changes in the Gini coefficient with and without revenue recycling. In particular, we analyse recycling via reductions in value-added tax (VAT) rates on fruits and vegetables and via uniform lump-sum transfers to every consumer, which has been demonstrated to reverse the regressive impact of a tax in the context of carbon pricing^16,17^.

## Results

### Meat expenditure patterns across quintiles

**Fig. 1:**
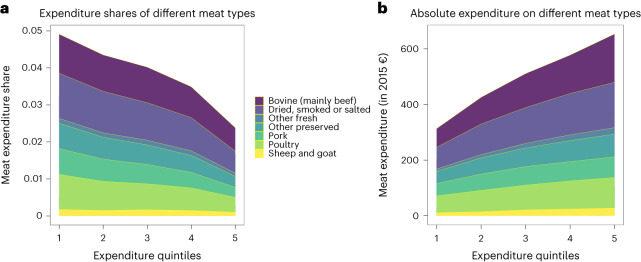
Relative versus absolute annual meat expenditure by meat type. **a**, The share of total expenditure spent on meat products across expenditure quintiles (Engel curve). **b**, The absolute expenditure on meat (in 2010 €) across quintiles. Each graph represents the country-weighted EU aggregate of country-level EU-HBS data. Source data

Assessing consumption patterns, the share of spending on meat (as a percentage of total expenditure) provides an intuition for the distributional effects of meat taxation. For the country-weighted EU average (Fig. 1), per capita spending on meat conforms with the known dynamics of Engel’s Law^18^: relative spending decreases across expenditure quintiles (Fig. 1a). Meanwhile, absolute annual spending increases with expenditure (Fig. 1b). This effect differs by meat type and appears to be more pronounced for beef than for sheep and goat, for instance. While most countries in the EU-HBS sample follow the described expenditure patterns, there are some exceptions. For example, the relative expenditure curve for France takes an inverted U shape, with meat expenditure shares relative to total expenses peaking among the middle class.

Overall, the above spending patterns—consumers in the lowest quintile spending a larger share of their budget on meat, while absolute expenditure increases for higher quintiles—suggest that meat taxation is regressive and can be turned progressive on average with lump-sum rebates. However, several factors specific to meat consumption suggest quantitative (and perhaps also qualitative) differences in the distributional impact of different tax design options. First, consumption of meat and other food items has satiation levels. This suggests that the spread of consumption in units across quintiles is much lower than expenditure: the per-kilogram meat consumption of individuals in the highest quintile is 29% higher than that of individuals in the lowest quintile, while high-income individuals spend 81% more on meat. Second, relatedly, preferences for quality attributes result in high-income individuals spending higher unit prices than low-income consumers. Third, as culinary traditions vary, we observe consumers in many southern European countries spending a comparatively larger share of their total expenditure on resource- and emissions-intensive beef and consumers in eastern European countries spending more on pork and meat products in the category ‘dried, salted and smoked’ compared to the average of the European sample (Supplementary Information).

### Distributional effects of different meat tax reforms

We assess the distributional impacts of taxing meat by analysing four main scenarios: (1) a 5% ad valorem tax on all meat types; (2) a shift from the reduced to the full VAT rate for all meat types; (3) a 50€ t^−1^ CO_2_ equivalent (CO_2_e) carbon tax based on the average GHG emissions (with the global warming potential of different GHGs expressed in equivalent amounts of carbon dioxide) associated with a specific meat type (that is, type of livestock); (4) a 0.35€ kg^−1^ unit tax on meat (that is, not differentiated by meat type), which is set such that it is equivalent to the 5% tax increase in scenario 1 in terms of total revenue. In each scenario, we compare three cases regarding the handling of the tax proceeds: in the first case, the tax revenues disappear (into the government budget); in the second case, the tax proceeds are returned to consumers as equal per capita payments; and in the third case, the revenue is used to reduce VAT rates on fruit and vegetables. A fourth case in which the revenue is used for targeted transfers to the lowest quintile is analysed in Supplementary Section 4 and Supplementary Table 3.

**Fig. 2:**
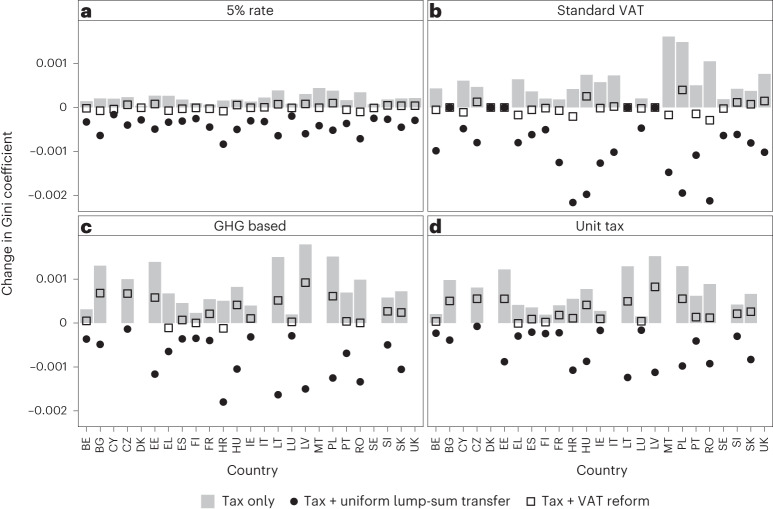
Simulated changes in post-reform Gini coefficients for four meat tax scenarios with revenue recycling via uniform lump-sum transfers and VAT reduction on fruit and vegetables. **a**, A 5% ad valorem tax on meat. **b**, An increase in VAT on meat to standard VAT rate. **c**, GHG-based meat taxes based on average per kg CO_2_e intensities of different meat types (beef, sheep and goat, pork, poultry and composite categories; Table 2) and a tax rate corresponding to 50€ t^−1^ CO_2_e. **d**, A 0.35€ kg^−1^ unit tax on meat. Grey bars indicate the effect of the tax only. Black dots indicate the net effect of the tax plus uniform lump-sum recycling, and squares indicate the net effect of the tax plus VAT reductions on fruit and vegetables. Country codes used are as indicated in the EU-HBS, which follow ISO 3166 alpha-2 (https://www.iso.org/iso-3166-country-codes.html), except for Greece, which is labelled EL, and the United Kingdom, which is labelled UK. Source data

On the basis of the EU-HBS, we conduct a microsimulation to determine the absolute per capita tax burden in each country and calculate the corresponding Gini coefficients in expenditure at the country level before and after the tax reform. We assume a demand elasticity of zero for the main results reported in Fig. 2 and Table 1 to determine the upper bound of the distributional effects. We check for robustness of our results using transferred elasticities from the literature in Supplementary Information. Average GHG intensities of different meat types used for this analysis are summarized in Table 2. For more details, see Methods section at the end of this paper.

**Table 1:** Summary of microsimulation results (changes in Gini coefficient and absolute annual per capita burden) for the population-weighted EU average

		(1) 5% rate	(2) Standard VAT	(3) GHG based	(4) Unit tax
Tax only	Change in Gini coefficient	0.000201 (0.000196, 0.000206)	0.000603 (0.000586, 0.000619)	0.000778 (0.000760, 0.000796)	0.000636 (0.000624, 0.000649)
	Absolute burden (1st quintile, €)	25.4 (25.0, 25.8)	73.1 (71.9, 74.3)	71.8 (68.8, 74.9)	54 (52.0, 56.0)
	Absolute burden (5th quintile, €)	48.6 (47.8, 49.4)	137.6 (135.2, 140.0)	92.7 (90.3, 95.1)	63.1 (61.8, 64.3)
Tax + uniform lump-sum transfer	Change in Gini coefficient	−0.000396 (−0.000402, −0.000390)	−0.00111 (−0.001128, −0.001093)	−0.00069 (−0.000709, −0.000671)	−0.00047 (−0.000482, −0.000458)
	Absolute burden (1st quintile, €)	−13 (−13.4, −12.6)	−36.2 (−37.4, −35.0)	−13.3 (−16.4, −10.3)	−6.4 (−8.4, −4.4)
	Absolute burden (5th quintile, €)	10.2 (9.4, 11.0)	28.2 (25.8, 30.6)	7.5 (5.2, 9.9)	2.6 (1.4, 3.9)
Tax + VAT reform	Change in Gini coefficient	0.000002 (−0.000004, 0.000007)	0.00003 (0.000014, 0.000047)	0.000241 (0.000220, 0.000261)	0.000238 (0.000224, 0.000252)
	Absolute burden (1st quintile, €)	1.1 (0.7, 1.5)	3.6 (2.4, 4.8)	14.9 (11.8, 18.0)	13.5 (11.4, 15.5)
	Absolute burden (5th quintile, €)	−3.5 (−4.4, −2.7)	−11.1 (−13.6, −8.5)	−19.5 (−22.5, −16.5)	−16.6 (−18.5, −14.8)

Absolute burden refers to the annual per capita burden in 2010 €. Negative values correspond to gains.

Figure 2 and Table 1 display the distributional outcome of the four scenarios as changes in the Gini coefficient. It is the standard economic measure of statistical dispersion to depict the distribution of income, or here expenditure, within a country—with 0 reflecting a state of perfect equality and 1 representing the most unequal state possible. Compared to the overall level of inequality in expenditure (that is, the Gini coefficients of total expenditure in the different countries ranging between 0.269 to 0.384 before taxes), the overall effect of meat taxation on inequality is small. For a more intuitive illustration of the magnitude of the effects of the reform, Table 1 additionally depicts the annual per capita burden of the tax reform in euros on the first and the fifth quintiles. For instance, the 0.0002 increase in the Gini coefficient corresponding to the first scenario without revenue recycling would lead to an annual per capita tax burden of 25.4€ on consumers in the lowest and 48.5€ on consumers in the highest quintile. This corresponds to 0.24% and 0.11% of total expenditure for the first and the fifth quintiles, respectively (Supplementary Section 2). We report 95% confidence intervals in Table 1 and find that the results are statistically significant in both metrics (Gini coefficient and absolute burden), both at the aggregate EU level and the national level (Supplementary Data).

The impact of meat taxation without recycling (grey bars in Fig. 2) is regressive across all four scenarios, while the degree of regressivity differs across scenarios and countries. This is primarily as the selected tax designs imply different levels of stringency. Whereas the 5% ad valorem tax (1) implies equal stringency of the tax reform across countries, a Europe-wide decision to increase VAT on meat to standard rates (2) implies different stringencies (equivalent to a 9–21% ad valorem tax increase), while five countries (Bulgaria, Denmark, Estonia, Latvia and Lithuania) already have standard VAT on meat products (Supplementary Table 5). The two unit tax scenarios (3) and (4) are constrained to a slightly smaller sample of countries due to a lack of data on corresponding meat quantities.

Recycling of meat tax revenues to consumers changes the distributional outcomes substantially. Total revenue raised in all 25 countries in scenarios (1) and (2) amounts to 6.1 billion € and 17.4 billion €, respectively. Scenarios (3) and (4) use a smaller sample of 19 countries. Here total revenue equals 5.2 billion € and 3.7 billion €, respectively. When revenues are redistributed to consumers as equal per capita transfers (indicated by black dots in Fig. 2), the distributional impact of a meat tax is rendered progressive in all four scenarios and in all countries. This outcome can be seen as a direct consequence of the Engel’s Law pattern (that is, decreasing relative meat expenditure and increasing absolute expenditure with increasing income in the majority of the analysed countries). When revenue is recycled via VAT rate reductions for fruit and vegetables (indicated by squares in Fig. 2), a similar logic applies. However, the regressivity mitigating effect is considerably lower: on the one hand, relative spending on fruit and vegetables follows the typical Engel’s law dynamic. Hence, reducing VAT rates on these goods will generally have a progressive effect. On the other hand, richer individuals spend more in absolute terms on these goods. Thus, they will benefit more in absolute terms. This dampens the progressive effect (as measured by the Gini coefficient). In sum, VAT reductions still dampen the regressivity of meat taxes in most cases, albeit to a much lesser extent than equal per capita transfers.

Moreover, we find that the distributional effect varies with tax design. The overall regressivity of the reform is reduced as one moves towards differentiating tax rates according to the average carbon intensity of meat types. On average, it reduces by around two-thirds as one moves from unit taxation to ad valorem taxes. However, the magnitude of these effects is only of second-order importance compared to the distributional effect of revenue recycling (Table 1). This is due to income-related expenditure patterns, in particular, the slightly higher carbon intensity and higher per-kilogram price of meat consumption among high-income individuals (Supplementary Figs. 5 and 6). More precisely, high-income individuals seem to consume more expensive meat products (for example, luxury cuts such as fillet or organically reared meat) and emissions-intensive types of meat (for example, beef), while low-income individuals consume cheaper meat products (for example, ground meat), on average.

**Fig. 3:**
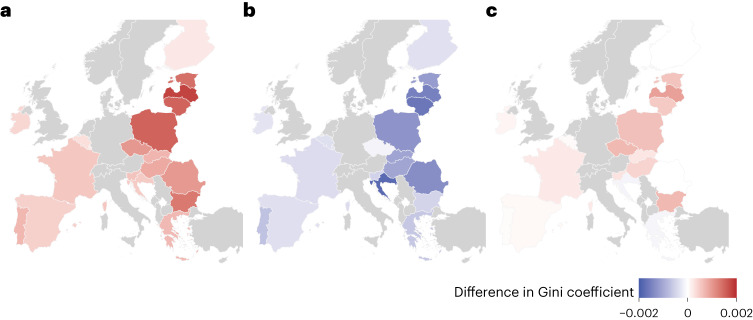
Simulated change in post-reform Gini coefficient for a GHG-based meat tax equivalent without redistribution of revenues, with revenue recycling via uniform lump-sum transfers and VAT reduction on fruit and vegetables. **a**, Results without redistribution of tax proceeds. **b**, The total effect when tax proceeds are redistributed through uniform lump-sum transfers. **c**, The total effect when tax proceeds are redistributed through lower VAT on fruits and vegetables. Source data

Although all scenarios are initially regressive when revenue recycling is not considered, the average annual per capita tax burden in the lowest quintile in absolute terms is still comparatively low (Fig. 3 for the GHG-differentiated tax scenario for each country and Supplementary Data for a summary of all scenarios). For instance, in the GHG-differentiated tax scenario, the tax burden in the lowest quintile ranges between 21€ and 98€ per year across the European Union with a population-weighted average of 72€.

We find that these results are very robust to assumptions about behavioural responses of consumers to tax increases and to the underlying dataset used. Regarding behavioural responses, for the main results summarized in Figs. 2 and 3, we assume that consumers do not adjust their spending patterns in response to the tax, that is, the price elasticity is assumed to be zero. This assumption serves to capture short-run distributional effects, which probably represent an overestimation: assuming a uniform price elasticity bigger than zero leads to qualitatively similar results, albeit with the distributional effects being of smaller magnitude (Supplementary Information). Regarding the robustness to the underlying data, we additionally perform the analysis using the 2015 wave of the EU-HBS. Although this dataset covers a smaller sample of countries for the variables we are interested in, the results confirm our main findings derived with the 2010 wave of the EU-HBS. These findings are detailed in Supplementary Table 2. In addition, the results remain qualitatively unchanged also when using much higher tax rates on meat that reflect the full social costs associated with meat consumption (Supplementary Fig. 4). Finally, we also check for robustness with regard to the inequality indicator used. We find that the results hold for the Theil index of expenditure when looking at the tax burdens of the different quintiles relative to income (Supplementary Table 5).

## Discussion

Simulating changes in consumer expenditure in response to different meat tax scenarios across a sample of 25 European countries (European Union and United Kingdom), we show that the regressive burden of meat taxes is mild for reasonably moderate tax levels. Moreover, the regressivity can be reversed in most cases even by non-targeted revenue recycling via uniform per capita transfers. Transfers targeted to the lowest quintile are, unsurprisingly, highly progressive and reduce inequality far beyond its pre-tax level. Revenue recycling via reductions in the VAT rate on fruit and vegetables dampens but in many countries does not fully offset the regressive effect of the tax. This leads to a roughly neutral distributional outcome in the ad valorem tax scenarios ((1) and (2)) and to a regressive outcome in the unit tax scenarios ((3) and (4)). Sensitivity analyses show that this mitigating effect of VAT reductions is further reduced with increasing demand elasticities and when tax rates increase (Supplementary Information). Our results imply that aside from varying stringency, revenue recycling is the strongest lever to reduce inequality impacts from meat taxation. The favourable distributional dynamic of moving towards ad valorem taxation is of second-order magnitude.

The results are very robust to assumptions regarding the behavioural response to the tax and the year of the underlying dataset. Nevertheless, our methods and data are subject to several caveats. First, due to a lack of robust meta studies on cross-price elasticities, we did not include substitution effects across meat types. While cross-price elasticities are usually estimated to be notably lower than own-price elasticities, substitution towards other meat types and cheaper cuts may nevertheless hamper the overall reduction in meat demand in response to a meat tax. Second, while a 100% tax incidence on consumers is a reasonably conservative assumption for assessing distributional effects, it will probably not hold up to reality. Imperfect market structures and complex pricing schemes—resulting, among other factors, from market power and price psychology—introduce some unpredictability as to how prices will adjust. Relatedly, regarding demand shifts between different cuts of the same meat type, it must be taken into account that all cuts have to be produced jointly in roughly fixed proportions. It is not obvious how producers, who need to maintain carcass balance, would respond to the introduction of a tax. Finally, horizontal inequalities and general equilibrium effects of meat taxation constitute promising areas for future research. Exploring consumer heterogeneity within income brackets^19,20^ can help identify more clearly which socioeconomic characteristics apart from income predict emissions-intensive dietary habits and a disproportionate burden from meat taxation. With respect to general equilibrium effects, for example, a tax-induced shift towards less GHG-intensive meat production such as lab-grown meat^21^ or a decreasing labour intensity could both influence the overall distributional outcome.

Moreover, the distributional implications are only one aspect for policy choice and the meat tax schemes discussed here may fare very differently in terms of their environmental effectiveness. Following Pigouvian principles, taxing pollutants directly instead of levying taxes on final product consumption, has a much higher steering effect, especially given the large variation in emissions across farms^6^. When opting for consumption taxes on meat, levying taxes based on total units instead of ad valorem and differentiating tax rates according to average GHG intensities can be presumed to be more effective in reducing emissions^15^. While ad valorem taxation of meat combines the appeal of simplicity with comparatively favourable distributional dynamics, it will probably incentivize quality substitution from high-value to low-value meat products. Aside from limiting emissions savings, this may result in increased consumption of comparatively unhealthier, highly processed products and meats from lower animal welfare standards. In addition, ad valorem taxation disadvantages high-value organic products, which fare better on at least some environmental indicators. In practice, these complexities might call for a more nuanced policy package with either tax exemptions or complementary subsidies for meats farmed under improved environmental and animal welfare standards. Such policy packaging has been shown to enhance the public support and thus the likelihood of adopting market-based food policies^8^. Furthermore, where policy packages are being implemented, distributional analyses should account for the inequality impacts of the package as a whole, rather than focusing on specific measures. This also applies to interactions with policies that are not exclusively focused on meat consumption but have some regulatory overlap, such as carbon taxes.

The policy choice between different design options for meat taxes is constrained by existing regulatory landscapes and questions of administrative implementability and political feasibility^22^. For example, due to large variation in existing VAT regulations across the European Union, levying the standard VAT rate on meat would result in price hikes as different as 0% for the Baltic countries and Bulgaria, which already tax meat at standard VAT rate, and 21% in Ireland, which currently levies no VAT on meat and other foodstuffs. The implementation of uniform lump-sum recycling of meat tax revenues is also likely to face several challenges: for one, policymakers might regard the modest amount of tax proceeds to be unworthy of the administrative effort of uniform redistribution, unless the reform can be implemented on the back of existing reforms, such as climate dividends or energy allowances. In addition, there may be political pressure for more targeted transfers to alleviate the burden on livestock farmers and producers along the supply chain. The fact that those on low incomes are expected to react more strongly to meat price increases^23^ might further strengthen the perception that the burden of the dietary shift is unfairly distributed. Therefore, more targeted transfers to low-income groups might be required to ensure feasibility. Unsurprisingly, targeted transfers to the lowest expenditure quintile (Supplementary Table 3) render the total reform even more progressive than uniform lump-sum redistribution.

Overall, our analysis indicates that policymakers in the European Union may introduce a consumption tax on meat without fearing adverse distributional outcomes between consumers in different expenditure quintiles as long as revenues are returned to citizens as uniform lump-sum payments. In addition, we show that the frequently debated option of using the revenues from a meat tax to finance reductions in the VAT rate on fruit and vegetables is generally not sufficient to reverse the regressive effect of the tax and leads to roughly distribution-neutral outcomes in the ad valorem tax scenarios ((1) and (2)) and to regressive outcomes in the unit tax scenarios ((3) and (4)).

## Methods

### Data on household expenditure, population and VAT rates

Our main source of information on household expenditure and consumption patterns is the EU-HBS. The EU-HBS’s primary purpose is to provide data for the construction of consumption baskets of goods to be used for consumer price indices. In an anonymized form, the microdata from the survey can be requested from Eurostat for research purposes. Our main analysis relies on the 2010 wave, which contains more data on the relevant consumption categories than the recently released 2015 wave (Supplementary Information). In terms of geographical coverage, our dataset spans the 27 Member States of the European Union as of 2020 (EU27) and the United Kingdom with the exception of Austria, Germany and the Netherlands, which do not provide sufficiently detailed data for our analysis. The 2015 sample additionally lacks sufficient data on Malta, Portugal, Slovenia and the United Kingdom.

For every sample household, the EU-HBS reports spending on different categories of goods and services over a specified period of time (typically two weeks) along with a variety of socioeconomic characteristics. Expenditures are categorized according to the Classification of Individual Consumption by Purpose. The Classification of Individual Consumption by Purpose headings relevant to our analysis are: Meat (01.1.2); Beef and veal (01.1.2.1); Pork (01.1.2.2); Sheep and goat (01.1.2.3); Poultry (01.1.2.4); Dried, salted or smoked meat and edible meat offal (01.1.2.5); Other preserved or processed meat and meat preparations (01.1.2.6); Other fresh, chilled or frozen edible meat (01.1.2.7); Fruit (01.1.6) and Vegetables (01.1.7). Spending in restaurants and canteens is not disaggregated into meat categories in the EU-HBS and therefore not included in the analysis. From the sample data, population-level estimates are calculated using the appropriate sampling weights as recommended by Eurostat, and the modified equivalence scales of the Organisation for Economic Co-operation and Development (OECD) accounting for economies of scale as the number of household members increases. As such, the unit of analysis is the individual, sometimes also referred to as the equivalized household. Quintiles are computed at the national level by ranking equivalized households (individuals) by their equivalent expenditure, that is, their total expenditure divided by the household size according to the modified OECD equivalence scale. EU-level results are computed based on population-weighted averages of national data.

In addition, while most member states collect information on consumer purchases in both monetary and physical terms, some only provide information on prices (Cyprus, Denmark, Italy, Malta, Sweden and the United Kingdom). These countries are thus excluded from scenarios (3) and (4), where quantities are needed to calculate the tax burden. The 2015 sample provides insufficient data on Bulgaria, Cyprus, France, Ireland, Italy, the Netherlands and Sweden.

We use official Eurostat databases for 2010/2015 population numbers and the European Commission’s overview for VAT rates (Supplementary Table 5).

### Data on the price elasticity of meat

There are several meta-analytical studies on the own-price elasticity of meat: Gallet^24^ predicts the own-price elasticity of composite meat to be around −0.850 based on a large sample of published studies. Price responsiveness is found to vary across species categories, with beef (−0.986) and lamb (−1.062) characterized by larger elasticities than both pork (−0.914) and poultry (−0.779), which exhibits the lowest elasticity. Andreyeva et al.^25^ do not report an elasticity for composite meat and find slightly lower elasticities for the individual meat categories (beef: −0.75, pork: −0.72 and poultry: −0.68). Slightly lower meat price elasticities are also found in ref. ^26^, which argues that the degree of product aggregation can play an important role in the analysis: studies that focus on narrowly defined meat product categories tend to find higher own-price elasticities (0.66 on average) than those that consider a general meat aggregate (0.5). Indeed, the broader the definition of a product, the greater is the scope for substitution within product category, rather than away from it.

Meta-analyses also suggest that meat demand becomes less responsive to price at higher income levels, both at country and consumer levels^23^. Further, the own-price elasticity of meat demand varies hugely from one study to another, reflecting differences in the nature of input data, econometric model specification and the estimation method^27^. It is thus not surprising that when comparable meta-analyses were attempted on cross-price elasticities—which generally have much smaller magnitudes than own-price elasticities—identifying any robust patterns proved difficult^28^.

In the analysis of GHG-equivalent food taxes, demand response models are often parameterized using estimates retrieved from the above-discussed meta-analyses^29,30^. Notable exceptions are studies of Spain^13^ and Germany^15^, which estimate elasticities ad hoc from household budget survey data, and France^31^, where panel data are used. In general, however, the results are close to the values identified by Gallet^24^.

Due to the incomplete sample for meat quantities in the EU-HBS, which is a prerequisite for estimating a demand system ad hoc, we rely on the elasticity estimates from Gallet^24^. They are based on a large sample and lie somewhere in the middle between higher estimates reported in ref. ^31^ and ref. ^13^ and lower estimates as used, for instance, in ref. ^14^. We check for robustness with the higher elasticities reported in (ref. ^31^) (2018; beef = −1.336; pork = −1.124; poultry (chicken) = −1.452; lamb/veal = −1.585; meat (aggregate) = −1.374.). The case of lower elasticities is covered by the simulation in the main part, which assumes completely inelastic demand.

### Data on GHG intensities and other environmental impacts of meat

GHG intensities used in earlier analyses of meat taxes in Europe display a non-negligible amount of variation. In the case of beef, for example, they range from 13.9 (ref. ^32^) to 25.1 (ref. ^13^) kg of CO_2_e per kg of meat. Similarly, values range between 5.3 (ref. ^31^) and 10.3 (ref. ^13^) kg of CO_2_e per kg of meat for pork and between 4 (ref. ^33^) and about 7 (ref. ^31^) kg of CO_2_e per kg of meat for poultry. While this variation may reflect genuine differences between the countries on which the different analyses focus, it seems likely that methodological differences among the underlying life-cycle assessments, for example, concerning system boundaries or reference units, also play a role.

This Article uses GHG intensities computed for the European Union by Weiss and Leip^34^ under their scenario II—the intermediate scenario in terms of GHG emissions from land use. GHG intensities are given for beef, poultry, sheep and goat and pork. For the categories for which we do not have GHG intensities (dried, salted and smoked, other preserved and processed, other meat), we estimate the GHG intensity as the average of the GHG intensities of the other meat types weighted by their consumption share at the EU level. The GHG intensities reported in Table 2 refer to a cradle-to-farm-gate life-cycle analysis and use kg of carcass meat as the reference unit.

**Table 2:** Average GHG intensities of different meat types in Europe

Meat type	CO_2_e (kg per kg meat)
Beef	22.5
Sheep and goat	20
Pork	7.5
Poultry	5
Other	13.0

Average emissions content in CO_2_e based on scenario II in Weiss and Leip^34^.

### Microsimulations

We conduct a microsimulation to analyse the four meat tax scenarios outlined above by determining the absolute per capita tax burden on each quintile in each country and calculating the corresponding Gini coefficients in expenditure at the country level before and after the tax reform. Specifically, the Gini coefficient after tax is calculated on the consumption expenditure after tax minus the cost of the tax plus eventual lump-sum transfers or gains via reduced VAT rates on fruit and vegetables, assuming that consumption expenditure proxies living standards. The Gini coefficient allows us to depict the heterogeneity in distributional outcomes between countries with a single indicator. However, any type of nonlinear indicator of inequality loses some information in the aggregation process. It generally provides a less intuitive measure, which is why we also calculate the per capita tax burden in absolute and relative terms.

It is assumed that the tax is entirely passed on to consumers. In terms of demand responses, we assume a demand elasticity of zero for the main results reported in Fig. 2 and Table 1 to approximate the short-term effects of the meat tax. In terms of distributional impacts, assuming a demand elasticity of zero represents an upper bound, as elasticities have been shown to be non-zero and higher for lower-income groups. This is a common approach in the literature that assesses the distributional effect of carbon and energy taxation^35–37^. Due to several underlying issues with the EU-HBS data that do not allow for estimating a demand system, for the long-term effects, we rely on transferred elasticities from other studies, as is done in similar studies. The results with a demand response are reported in Supplementary Table 1 and are qualitatively the same but of lower magnitude.

For scenarios (1) and (2), only expenditure data are required. This allows for analysing a sample of 25 EU countries and a corresponding sample size of 220,885. For scenarios (3) and (4), additional data and the quantities of meat consumed are needed. This removes further six countries from the sample, which implies a smaller sample size of 136,998. Aggregation of quintiles and all outcomes are calculated at the country level. Whenever results are given at the EU level, they are calculated as population-weighted averages.

For the microsimulation results at the EU level, 95% confidence intervals are reported in Table 1. For readability, they are omitted from the figures. The calculations were carried out following Penne and Goedemé^10^. For each combination of country, scenario and revenue recycling scheme, we use the ‘digini’ function from the DASP module^38^ in Stata to compute the change in the Gini coefficient relative to the baseline and the associated standard error. Mean per capita burden estimates are obtained using built-in Stata functions for survey analysis.

## Disclaimer

The views expressed are purely those of the authors and may not in any circumstances be regarded as stating an official position of the European Commission.

### Reporting summary

Further information on research design is available in the Nature Portfolio Reporting Summary linked to this article.

### Supplementary information

Supplementary Information Supplementary Figs. 1–7, Discussion and Tables 1–5.

Reporting Summary

Supplementary Data Supplementary country-level microsimulation results.

### Source data

Source Data Figs. 1 and 3 Source data for Figs. 1 and 3.

Source Data Fig. 2 Source data for Fig. 2.

## Data Availability

Our main source of data is Eurostat’s Household Budget Survey (https://ec.europa.eu/eurostat/web/microdata/household-budget-survey). Researchers can request access to these data under specific conditions laid out in this document (https://ec.europa.eu/eurostat/documents/203647/771732/How_to_apply_for_microdata_access.pdf). Additional data on population are available through Eurostat’s Population and Democracy database (https://ec.europa.eu/eurostat/web/population-demography/demography-population-stock-balance/database). Data on VAT rates in the European Union are reprinted in Supplementary Table 5. Source data are provided with this paper.

## Code availability

The R and Stata code used for conducting the analysis is available at 10.5281/zenodo.8308085.
