# Supplementary material for: Meat taxes in Europe can be designed to avoid overburdening low-income consumers
Source: Nat Food. 2023 Oct 2;4(10):894–901. doi: 10.1038/s43016-023-00849-z (PMC10589082; doi:10.1038/s43016-023-00849-z)
Supplement: Supplementary file 1 — Supplementary Figs. 1–7, Discussion and Tables 1–5. [file 43016_2023_849_MOESM1_ESM.pdf]

nature food

Article

https://doi.org/10.1038/s43016-023-00849-z

Meat taxes in Europe can be designed to avoid overburdening low-income consumers

In the format provided by the authors and unedited

Supplementary Information

"Meat taxes in Europe can be designed to avoid overburdening low-income consumers"

1. Demand Response 2

Supplementary Figure 1: Comparison of policy scenarios with demand response. 3

Supplementary Table 1: Summary of results with demand response. 4

2. Analysis with 2015 data 6

Supplementary Figure 2: Relative vs. absolute meat expenditure (2015 data). 6

Supplementary Figure 3: Comparison of policy scenarios for 2015 data. 7

Supplementary Table 3: Summary of results with 2015 data. 8

3. Additional scenario: Tax based on environmental social costs of meat 9

Supplementary Figure 4: Distributional effects of meat tax with revenue recycling for a tax based on environmental social costs 9

4. Additional recycling pathway: Targeted transfers to the lowest quintile 10

Supplementary Table 4: Summary of results with targeted transfers 11

5. Alternative inequality measures: Theil index and relative tax burdens 12

Supplementary Table 5: Results with alternative inequality measures 12

6. Value Added Tax Rates 13

Supplementary Table 5: VAT rates in 2010 and 2015 in EU countries. 13

7. Meat price and GHG emissions by meat type and quintile 14

Supplementary Figure 5: Meat price per quintile and kilogram 14

Supplementary Figure 6: Annual GHG emissions per quintile and meat type 14

8. Relative spending by meat type at the country level 15

Supplementary Figure 7: Country-level Engel curves. 15

9. References 25

1. Demand Response

We also model the behavioural response of consumers to the different tax and rebate scenarios. For scenarios one and two, we use the own-price elasticity of the generic category 'meat' which encompasses all different meat types (-0.850). For scenarios three and four, we use specific own price elasticities of beef (-0.985), pork (-0.913), sheep and goat (-1.062) and poultry (-0.778), as described in the methodology section.

We find that all previous results hold qualitatively when accounting for a demand response by the consumers. However, the regressive impact of an uncompensated meat tax is diminished by a shift in consumption away from meat. This also implies that there is less tax revenue to redistribute and hence the progressive effect of the revenue recycling diminishes as well. This is particularly true for recycling via reductions in the VAT rate. The uniform lump-sum transfers, however, still offset the regressive effect of the tax and lead to an overall progressive outcome in the EU average and in all countries except for Ireland. The results are summarised in Supplementary Figure 1 and Supplementary Table 1.

We also checked the robustness of our findings using the relatively high elasticity estimates from Bonnet et al. (2018)[1]. The results from this analysis are shown in Supplementary Table 2. Similar to the case where we moved from a complete inelasticity of demand (Table 1 in the main manuscript) to an elastic demand with elasticities from Gallet (2010) (Supplementary Table 1), the results are qualitatively the same but distributional impacts of both the tax and the redistribution are further diminished with increasing elasticities.

[1] The elasticities from (Bonnet et al., 2018) are: beef = -1.336; pork = -1.124; poultry (chicken) = -1.452; lamb/veal = -1.585; meat (aggregate) = -1.374.

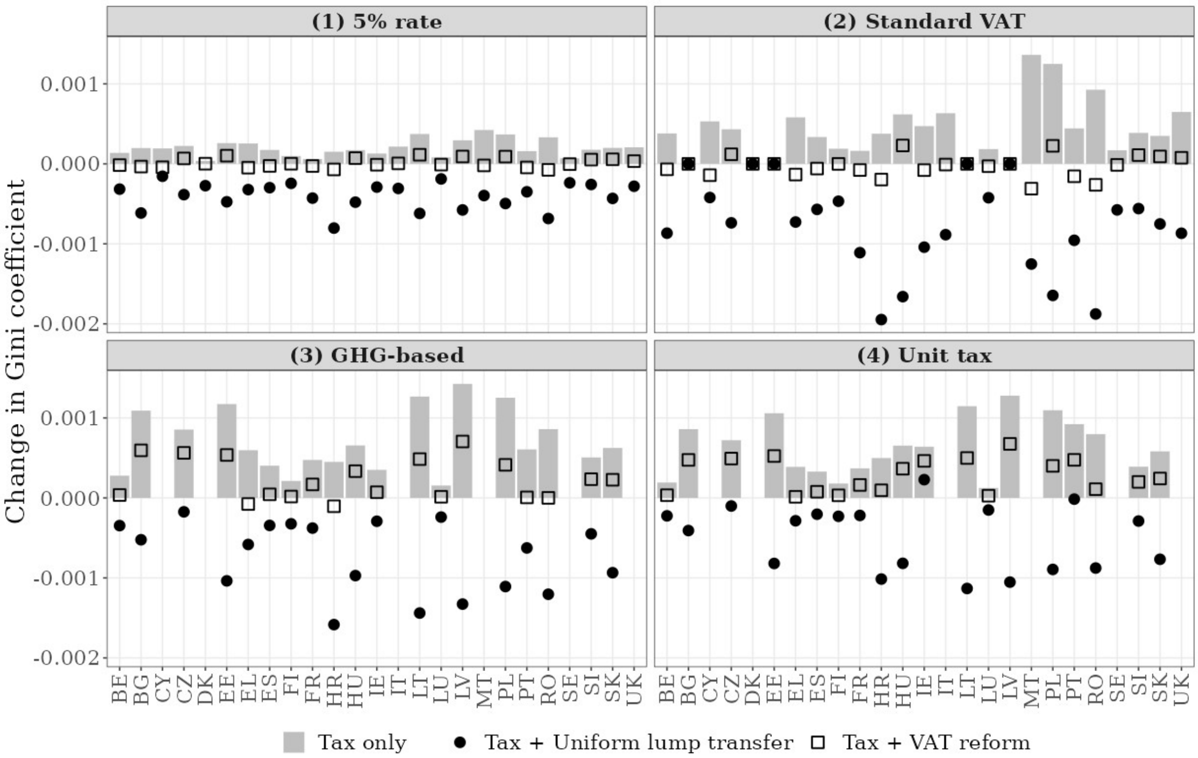

Supplementary Figure 1: Comparison of policy scenarios with demand response. *For 2010 EU-HBS Data. Panel (1): A 5% ad valorem tax on meat; Panel (2): Increasing VAT on meat to standard rate; Panel (3): GHG-based meat taxes (50€/tCO2e); Panel (4): A 0.35€/kg unit tax on meat. Grey bars indicate the effect of the tax only. Black dots indicate the net effect of the tax + uniform lump-sum recycling and squares indicate the net effect of the tax + VAT reductions on fruit and vegetables.*

Supplementary Table 1: Summary of results with demand response. *Elasticity estimates from the meta study by Gallet (2010). Results for the country-weighted EU average. Absolute burden refers to the annual absolute per-capita burden in 2010 €. Negative values correspond to gains.*

		**(1) 5% rate**	**(2) Standard VAT**	**(3) GHG-based**	**(4) Unit tax**
**Tax only**	Change in Gini coefficient	0.000193 (0.000188, 0.000198)	0.000521 (0.000507, 0.000535)	0.000665 (0.000649, 0.000681)	0.000585 (0.000575, 0.000596)
	Absolute burden (1st Quintile, €)	24.4 (24.0, 24.8)	63.9 (62.8, 64.9)	62.9 (60.4, 65.5)	49.2 (47.4, 50.9)
	Absolute burden (5th Quintile, €)	46.6 (45.8, 47.4)	120.5 (118.4, 122.6)	84 (81.9, 86.1)	59.1 (57.9, 60.3)
**Tax + Uniform lump transfer**	Change in Gini coefficient	-0.00038 (-0.000386, -0.000375)	-0.00097 (-0.000985, -0.000955)	-0.000632 (-0.000649, -0.000616)	-0.000423 (-0.000435, -0.000412)
	Absolute burden (1st Quintile, €)	-12.5 (-12.9, -12.1)	-31.8 (-32.9, -30.8)	-13.3 (-15.9, -10.8)	-6.8 (-8.5, -5.0)
	Absolute burden (5th Quintile, €)	9.8 (9.0, 10.5)	24.8 (22.7, 26.9)	7.8 (5.6, 9.9)	3.2 (2.0, 4.4)
**Tax + VAT reform**	Change in Gini coefficient	0.000006 (0.000000, 0.000011)	-0.000003 (-0.000018, 0.000012)	0.000184 (0.000166, 0.000202)	0.000223 (0.000210, 0.000235)
	Absolute burden (1st Quintile, €)	1.4 (1.0, 1.8)	-0.1 (-1.2, 1.0)	11.4 (8.8, 14.0)	11.9 (10.0, 13.7)
	Absolute burden (5th Quintile, €)	-2.6 (-3.4, -1.8)	-16.5 (-18.7, -14.2)	-17.5 (-20.2, -14.8)	-14.2 (-15.9, -12.5)

Supplementary Table 2*: Summary of results for the country-weighted EU average (including a demand reaction with high elasticity estimates from Bonnet et al., 2018)* *Absolute burden refers to the annual absolute burden in 2010 €. Negative values correspond to gains.*

		(1) 5% rate	(2) Standard VAT	(3) GHG-based	(4) Unit tax
Tax only	Change in Gini coefficient	0.000188 (0.000183, 0.000193)	0.000471 (0.000459, 0.000484)	0.000602 (0.000588, 0.000617)	0.00054 (0.000530, 0.000551)
	Absolute burden (1st Quintile, €)	23.7 (23.4, 24.1)	58.2 (57.2, 59.1)	58.3 (56.0, 60.6)	46.2 (44.6, 47.8)
	Absolute burden (5th Quintile, €)	45.4 (44.7, 46.2)	110 (108.1, 111.9)	79.8 (77.8, 81.8)	56.7 (55.5, 57.9)
Tax + Uniform lump transfer	Change in Gini coefficient	-0.000371 (-0.000376, -0.000365)	-0.000883 (-0.000897, -0.000869)	-0.000607 (-0.000622, -0.000591)	-0.000409 (-0.000419, -0.000398)
	Absolute burden (1st Quintile, €)	-12.2 (-12.5, -11.8)	-29.1 (-30.1, -28.2)	-13.5 (-15.8, -11.2)	-7 (-8.6, -5.4)
	Absolute burden (5th Quintile, €)	9.5 (8.8, 10.3)	22.7 (20.8, 24.6)	8 (6.0, 10.0)	3.5 (2.3, 4.7)
Tax + VAT reform	Change in Gini coefficient	0.000006 (0.000001, 0.000011)	-0.000001 (-0.000014, 0.000012)	0.000156 (0.000139, 0.000173)	0.0002 (0.000188, 0.000212)
	Absolute burden (1st Quintile, €)	1.4 (1.0, 1.8)	0.4 (-0.6, 1.4)	10 (7.7, 12.4)	10.9 (9.2, 12.6)
	Absolute burden (5th Quintile, €)	-2.5 (-3.3, -1.7)	-13.8 (-15.9, -11.7)	-15.3 (-17.9, -12.8)	-12.7 (-14.4, -11.1)

2. Analysis with 2015 data

Here we use the 2015 wave of the EU HBS. We have opted for using the 2010 wave for deriving our main results for the following reasons: First, consumption patterns have not shifted significantly between 2010 and 2015 in the EU and thus no additional insights are gained from the study of the 2015 data, except for the fact that our findings are robust to possible differences in the data. Second, in the 2015 wave, fewer countries report on the specific consumption categories that are relevant for our analysis and hence the sample is smaller.[2]

Relative and absolute expenditure patterns are shown in Supplementary Figure 2 and main results on the distributional effects without accounting for a demand response are displayed in Supplementary Figure 3 and Supplementary Table 3. The results are remarkably similar to the results derived from the 2010 wave of the HBS (see Figure 2 and Table 1 in the main manuscript): All four scenarios are progressive for all countries when the revenue is redistributed in a lump-sum fashion while recycling via VAT rate reductions is less effective in mitigating the regressive effect of the tax.

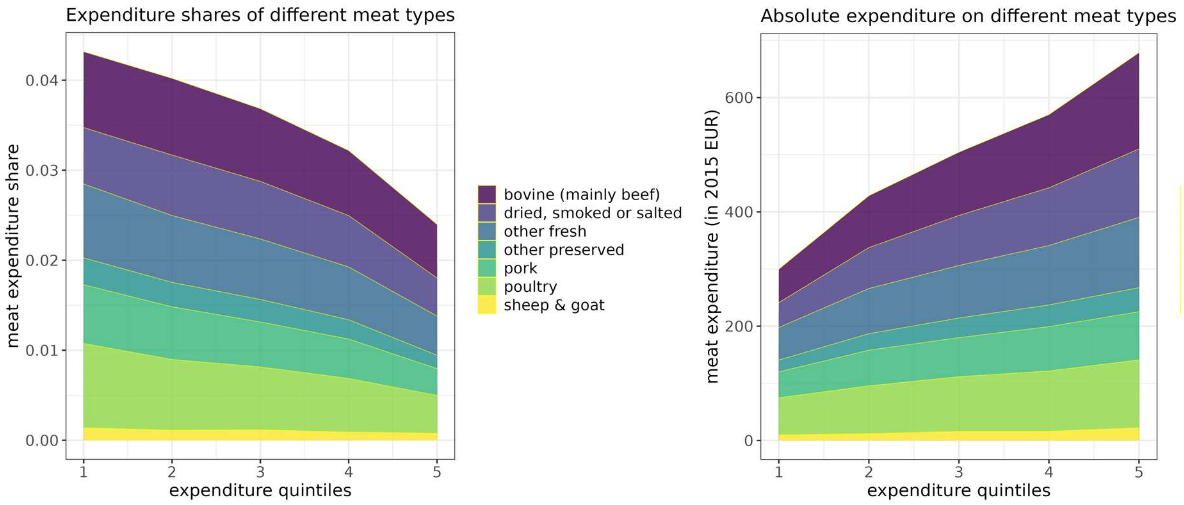

Supplementary Figure 2: Relative vs. absolute meat expenditure (2015 data). *Country-weighted EU average for annual expenditure by meat types.*

[2] In scenarios one and two there are 25 countries in the sample in 2010 and 22 in 2015. In Scenarios three and four, there are 19 countries in the 2010 sample and 13 in the 2015 sample.

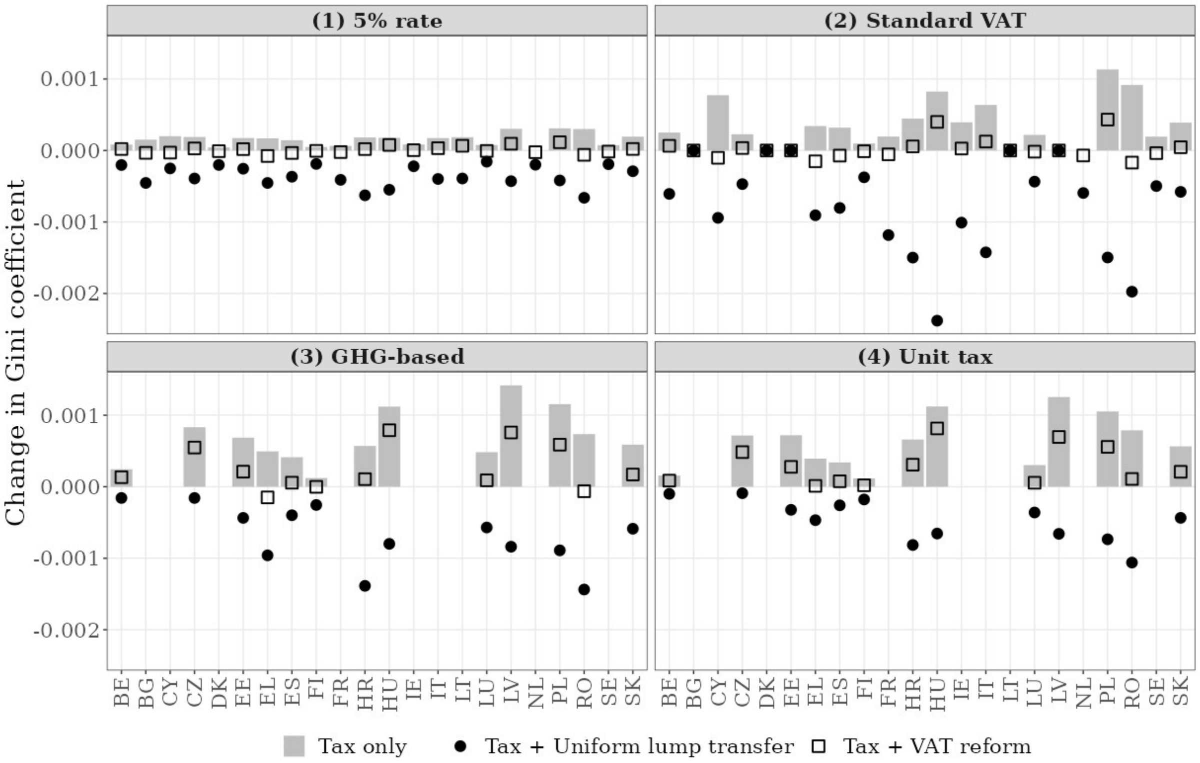

Supplementary Figure 3: Comparison of policy scenarios for 2015 data.

Without demand response. Panel (1): A 5% ad valorem tax on meat; Panel (2): Increasing VAT on meat to standard rate; Panel (3): GHG-based meat taxes (50€/tCO2e); Panel (4): A unit tax on meat. Grey bars indicate the effect of the tax only. Black dots indicate the net effect of the tax + uniform lump-sum recycling and squares indicate the net effect of the tax + VAT reductions on fruit and vegetables.

Supplementary Table 3: Summary of results with 2015 data. *Results for the country-weighted EU average. Absolute burden refers to the annual absolute per-capita burden in 2015 €. Negative values correspond to gains.*

		**(1) 5% rate**	**(2) Standard VAT**	**(3) GHG-based**	**(4) Unit tax**
**Tax only**	Change in Gini coefficient	0.000152 (0.000147, 0.000158)	0.000451 (0.000435, 0.000467)	0.000699 (0.000681, 0.000717)	0.00064 (0.000627, 0.000653)
	Absolute burden (1st Quintile, €)	24.5 (24.1, 25.0)	71.8 (70.4, 73.2)	57.8 (56.0, 59.7)	47.9 (47.0, 48.8)
	Absolute burden (5th Quintile, €)	48.2 (47.2, 49.1)	139 (136.3, 141.8)	74.8 (72.8, 76.8)	55.6 (54.3, 56.9)
**Tax + Uniform lump transfer**	Change in Gini coefficient	-0.00039 (-0.000395, -0.000384)	-0.001102 (-0.001120, -0.001085)	-0.000697 (-0.000715, -0.000679)	-0.000504 (-0.000516, -0.000491)
	Absolute burden (1st Quintile, €)	-12.6 (-13.1, -12.1)	-36.1 (-37.6, -34.7)	-9.1 (-10.9, -7.2)	-4.5 (-5.4, -3.6)
	Absolute burden (5th Quintile, €)	11 (10.1, 12.0)	31.1 (28.3, 33.8)	7.9 (5.9, 9.9)	3.3 (2.0, 4.6)
**Tax + VAT reform**	Change in Gini coefficient	0.000007 (0.000000, 0.000013)	0.000047 (0.000029, 0.000066)	0.000244 (0.000225, 0.000263)	0.000274 (0.000260, 0.000288)
	Absolute burden (1st Quintile, €)	2 (1.5, 2.4)	6.5 (5.0, 7.9)	12.1 (10.3, 13.9)	12.1 (11.2, 13.0)
	Absolute burden (5th Quintile, €)	-4 (-5.1, -2.8)	-12.8 (-15.9, -9.7)	-14.5 (-16.6, -12.4)	-14.4 (-15.8, -12.9)

3. Additional scenario: Tax based on environmental social costs of meat

In the following, we assess the distributional effects of taxing meat products in accordance with average social costs by meat type from GHG emissions (at a social cost of carbon of 100 USD/tCO_2e), and nutrient pollution from acidification and eutrophication (Funke et al. 2022). Due to scarce data on the environmental impacts at the product level and associated social costs, the damages from biodiversity loss are omitted. The scenario is qualitatively similar to the GHG-differentiated tax scenario, as climate impacts dominate the environmental social costs of meat, but notably more ambitious due to the higher carbon tax rate and inclusion of damages from nutrient pollution. Small variations in the GHG tax component apply as Funke et al. (2022) use global lifecycle data (Poore and Nemecek, 2018) rather than EU averages for GHG emissions contents.

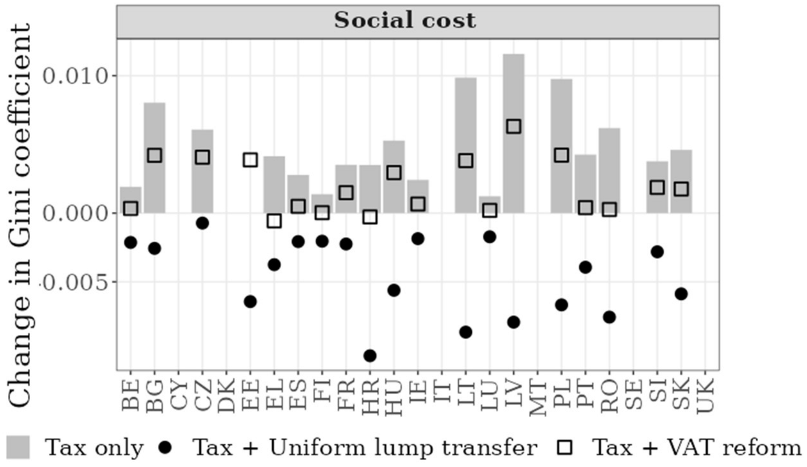

Supplementary Figure 4: Distributional effects of meat tax with revenue recycling for a tax based on environmental social costs*. For 2010 data, excluding a demand response.*

4. Additional recycling pathway: Targeted transfers to the lowest quintile

In this section, we analyse an additional scenario in which the tax revenue is used for targeted transfers to the lowest quintile. This scenario is only for illustrative purposes as in reality" such transfers would likely be dependent on the households' income sources and levels.

This revenue recycling pathway is the most progressive in all analysed scenarios. It reduces the Gini coefficient by a multiple of the reduction in the Gini coefficient induced by uniform lump-sum transfers (see Supplementary Table 4). The population weighted EU average of the targeted transfers to the lowest quintile can reach levels of up to 482 € per year.

Supplementary Table 4: Summary of results with targeted transfers*. Absolute burden refers to the annual absolute per-capita burden in 2010 €. Negative values correspond to gains.*

		(1) 5% rate	(2) Standard VAT	(3) GHG-based	(4) Unit tax
**Tax only**	Change in Gini coefficient	0.000201 (0.000196, 0.000206)	0.000603 (0.000586, 0.000619)	0.000778 (0.000760, 0.000796)	0.000636 (0.000624, 0.000649)
	Absolute burden (1st Quintile, €)	25.4 (25.0, 25.8)	73.1 (71.9, 74.3)	71.8 (68.8, 74.9)	54 (52.0, 56.0)
	Absolute burden (5th Quintile, €)	48.6 (47.8, 49.4)	137.6 (135.2, 140.0)	92.7 (90.3, 95.1)	63.1 (61.8, 64.3)
**Tax + Uniform lump transfer**	Change in Gini coefficient	-0.000396 (-0.000402, -0.000390)	-0.00111 (-0.001128, -0.001093)	-0.00069 (-0.000709, - 0.000671)	-0.00047 (-0.000482, - 0.000458)
	Absolute burden (1st Quintile, €)	-13 (-13.4, -12.6)	-36.2 (-37.4, -35.0)	-13.3 (-16.4, -10.3)	-6.4 (-8.4, -4.4)
	Absolute burden (5th Quintile, €)	10.2 (9.4, 11.0)	28.2 (25.8, 30.6)	7.5 (5.2, 9.9)	2.6 (1.4, 3.9)
**Tax + VAT reform**	Change in Gini coefficient	0.000002 (-0.000004, 0.000007)	0.00003 (0.000014, 0.000047)	0.000241 (0.000220, 0.000261)	0.000238 (0.000224, 0.000252)
	Absolute burden (1st Quintile, €)	1.1 (0.7, 1.5)	3.6 (2.4, 4.8)	14.9 (11.8, 18.0)	13.5 (11.4, 15.5)
	Absolute burden (5th Quintile, €)	-3.5 (-4.4, -2.7)	-11.1 (-13.6, -8.5)	-19.5 (-22.5, -16.5)	-16.6 (-18.5, -14.8)
**Tax + transfers to lowest quintile**	Change in Gini coefficient	-0.001560 (-0.001584, -0.001536)	-0.004342 (-0.004416, -0.004269)	-0.003541 (-0.003615, -0.003467)	-0.002612 (-0.002664, -0.002560)
	Absolute burden (1st Quintile, €)	-166.5 (-167.0, -166.1)	-473.6 (-474.8, -472.4)	-352.4 (-355.5, -349.3)	-247.0 (-249.0, -245.0 ()
	Absolute burden (5th Quintile, €)	48.6 (47.8, 49.4)	137.6 (135.2, 140.0)	92.7 (90.3, 95.1)	63.1 (61.8, 64.3)

5. Alternative inequality measures: Theil index and relative tax burdens

To check the robustness of our findings, in this section we additionally look at the Theil index and at the tax burden relative to total expenditure. The Theil index is a measure of inequality that ranges from 0 (perfect equality) to a maximum value of ln(N), where N is the number of households and ln is the natural logarithm. In the 2010 sample, the before tax Theil index of total expenditure ranges between 0.12 and 0.25. To get an idea of the scope of the changes in inequality, Table 1 from the main manuscript and Supplementary Table 5 can be compared. For instance, scenario 1 without revenue recycling leads to an absolute tax burden of 25€ (0.24% of their total expenditure) on the first quintile and 49€ (0.11% of their total expenditure) on the fifth quintile, which corresponds to an increase in the Theil index of 2.4e-4.

The results using the Theil index, and the relative tax burdens as shown in Supplementary Table 5 confirm our results with the Gini coefficient: All scenarios lead to an increase in the Theil index (i.e. more inequality), without revenue recycling. If the revenue is recycled via uniform lump-sum taxes, the Theil index decreases in all scenarios. Recycling via VAT cuts has largely negligible distributional effects for scenarios 1 and 2 and a slightly larger increasing effect on the Theil index in scenarios 3 and 4. Whenever we find a scenario and a recycling mechanism to be inequality-increasing in terms of the Gini coefficient and the Theil index, this is confirmed by the fact that the relative tax burden is (usually 2 to 3 times) larger for the lowest quintile, compared to the highest quintile. The opposite is true for the inequality-reducing scenarios.

Supplementary Table 5: Results with alternative inequality measures*. Relative burden indicates the tax burden relative to total expenditure. Negative values correspond to gains. All values correspond to the population weighted EU average.*

		(1) 5% ad valorem tax	(2) Increase to standard VAT	(3) GHG-differentiated unit tax (50€/CO2e)	(4) Flat unit tax (0.35€/kg)
**Tax effect**	Change in Theil index	0.000243	0.000734	0.000778	0.000636
	Relative burden (1st Quintile)	0.24%	0.69%	0.64%	0.47%
	Relative burden (5th Quintile)	0.11%	0.32%	0.23%	0.15%
**Tax + lump-sum transfers**	Change in Theil index	-0.000403	-0.00113	-0.000692	-0.000471
	Relative burden (1st Quintile)	-0.12%	-0.34%	-0.11%	-0.06%
	Relative burden (5th Quintile)	0.02%	0.07%	0.02%	0.01%
**Tax + VAT reform**	Change in Theil index	0.000005	0.000039	0.000240	0.000238
	Relative burden (1st Quintile)	0.01%	0.03%	0.12%	0.11%
	Relative burden (5th Quintile)	-0.01%	-0.03	-0.05%	-0.04%

6. Value Added Tax Rates

Supplementary Table 5: VAT rates in 2010 and 2015 in EU countries. *(EU Commission, 2020).*

Country	VAT meat 2010	VAT standard 2010	VAT meat 2015	VAT standard 2015
BE	6	21	6	21
BG	20	20	20	20
CZ	10	20	15	21
DK	25	25	25	25
DE	7	19	7	19
EE	20	20	20	20
IE	0	21	0	23
EL	11	23	13	23
ES	8	18	10	21
FR	5.5	19.6	5.5	20
HR	10	23	13	25
IT	4	20	4	22
CY	0	15	0	19
LV	21	21	21	21
LT	21	21	21	21
LU	3	15	3	17
HU	5	25	5	27
MT	0	18	0	18
NL	6	19	6	21
AT	10	20	10	20
PL	3	22	5	23
PT	6	21	6	23
RO	9	24	9	24
SI	8.5	20	9.5	22
SK	10	19	10	20
FI	13	23	14	24
SE	12	25	12	25
UK	0	17.5	0	20

7. Meat price and GHG emissions by meat type and quintile

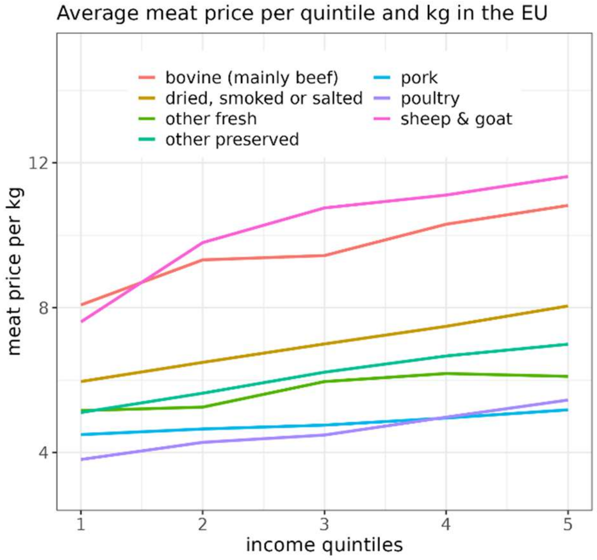

***Supplementary Figure 5: Meat price per quintile and kilogram**. Population-weighted EU average.*

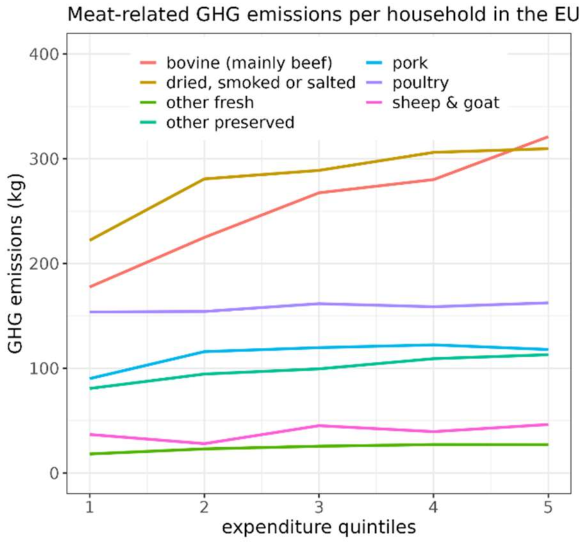

***Supplementary Figure 6: Annual GHG emissions per quintile and meat type**. Population-weighted EU average*

8. Relative spending by meat type at the country level

Supplementary Figure 7: Country-level Engel curves. *Share of expenditure on meat products relative to total expenditure.*

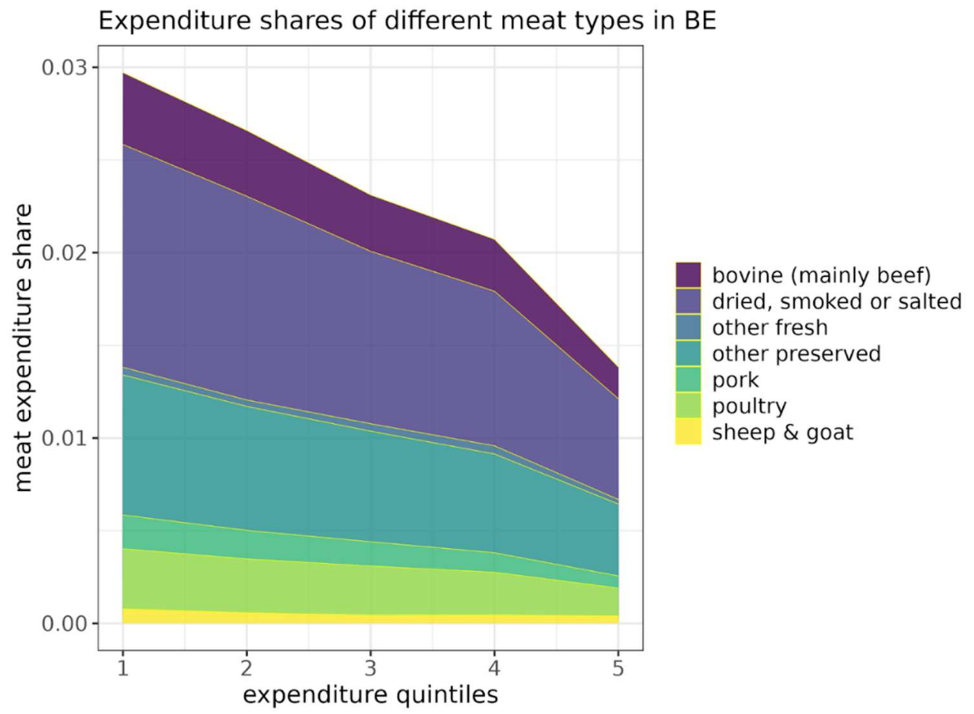

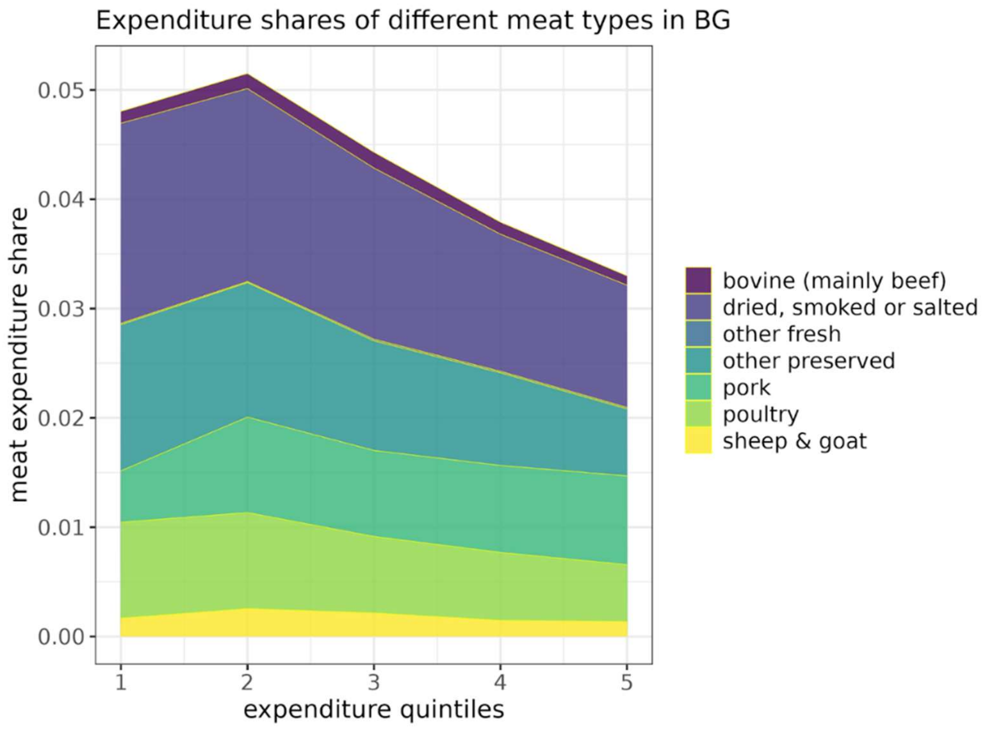

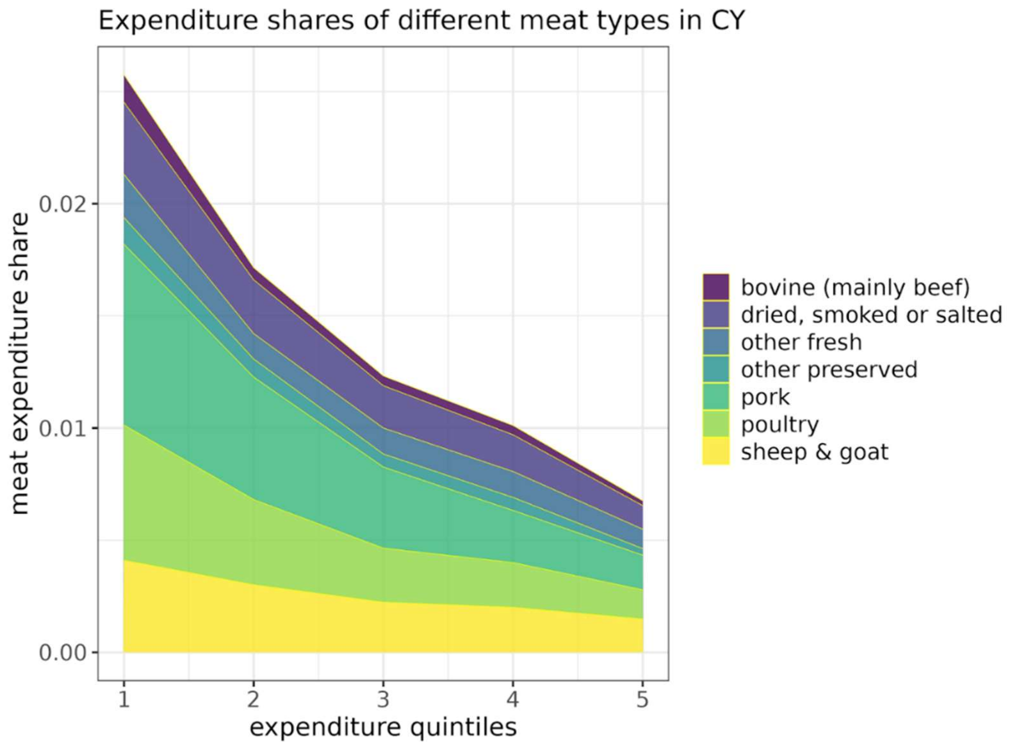
Expenditure shares of different meat types in CY
meat expenditure share
0.02
0.01
0.00
1
2
3
4
5
expenditure quintiles
bovine (mainly beef)
dried, smoked or salted
other fresh
other preserved
pork
poultry
sheep & goat

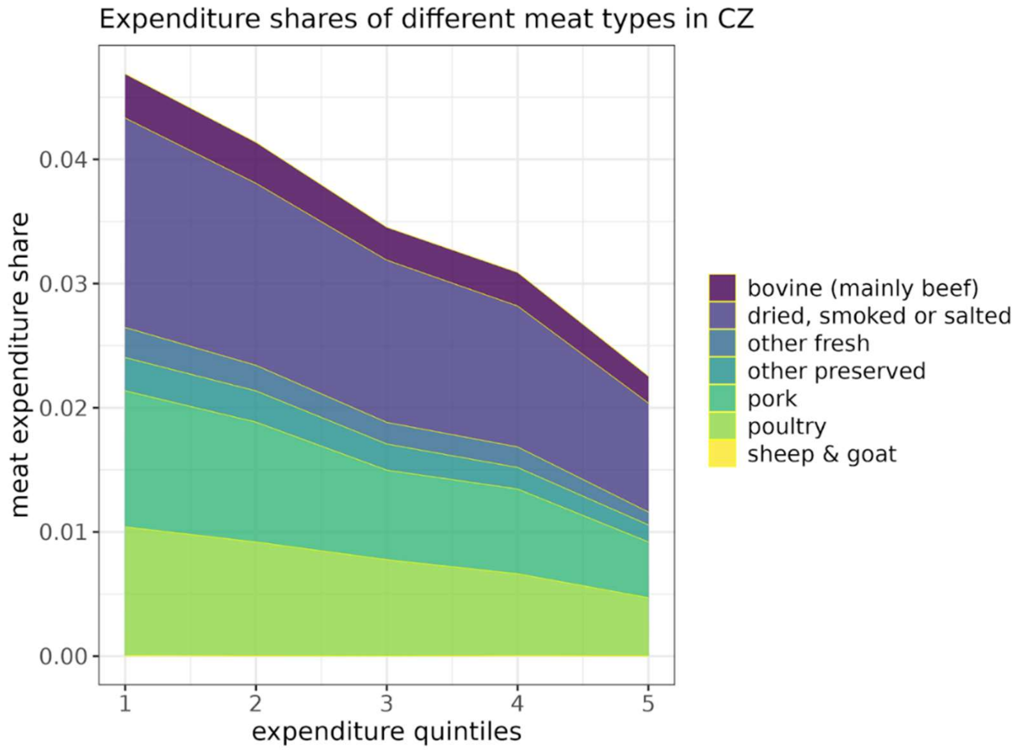
Expenditure shares of different meat types in CZ
meat expenditure share
0.04
0.03
0.02
0.01
0.00
1
2
3
4
5
expenditure quintiles
bovine (mainly beef)
dried, smoked or salted
other fresh
other preserved
pork
poultry
sheep & goat

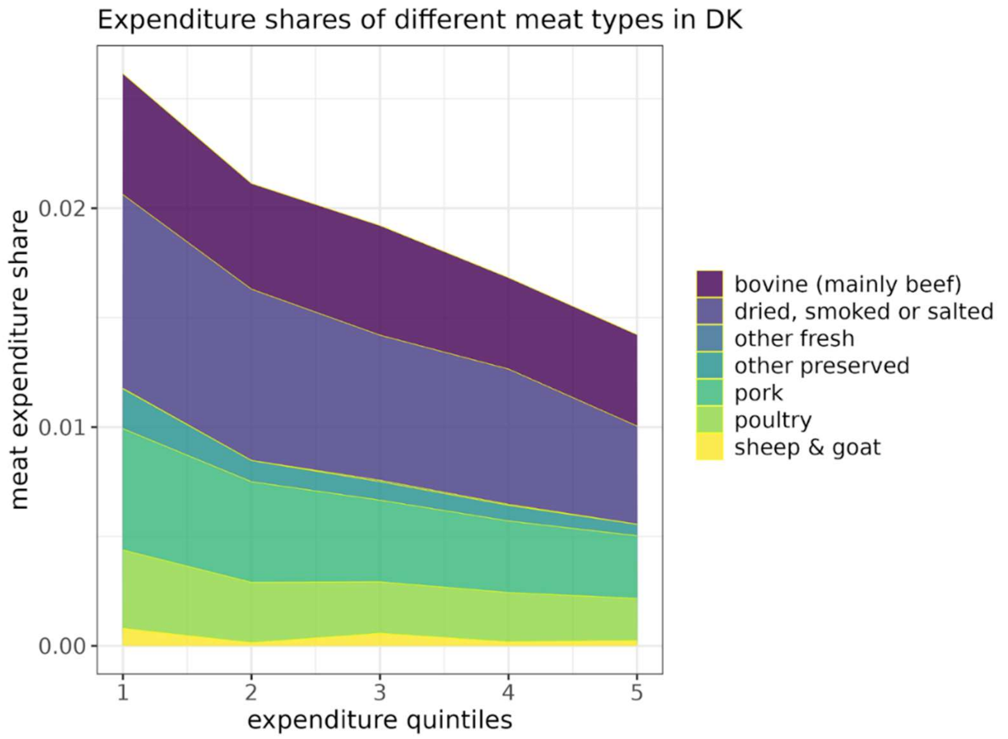
Expenditure shares of different meat types in DK
meat expenditure share
0.00
0.01
0.02
1
2
3
4
5
expenditure quintiles
bovine (mainly beef)
dried, smoked or salted
other fresh
other preserved
pork
poultry
sheep & goat

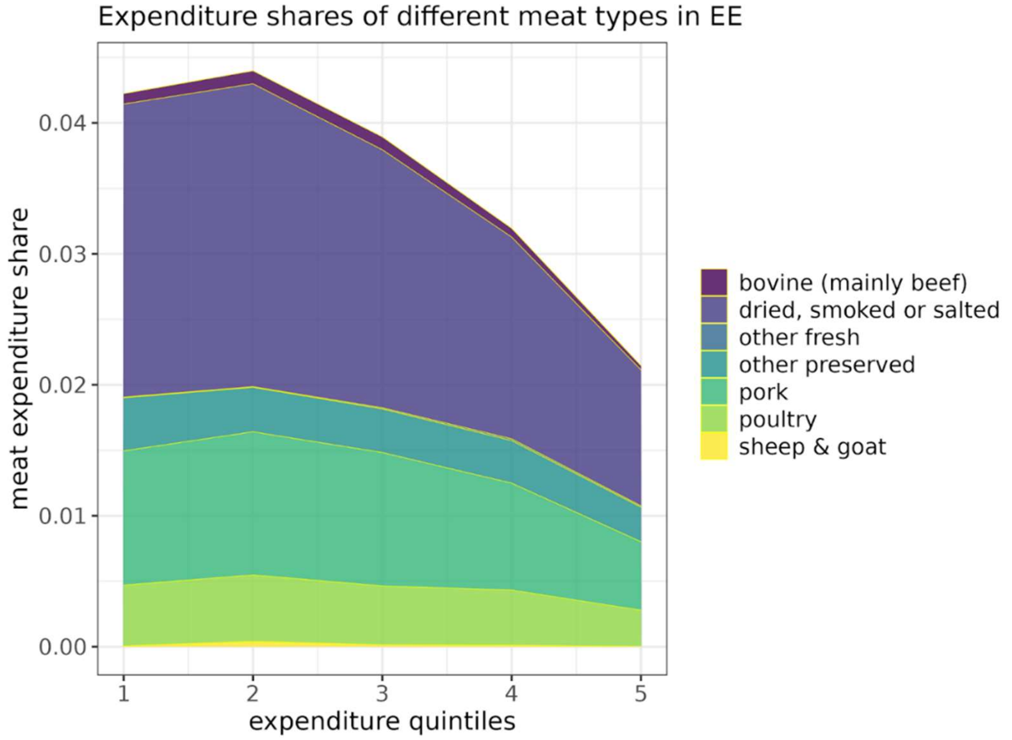
Expenditure shares of different meat types in EE
meat expenditure share
0.00
0.01
0.02
0.03
0.04
1
2
3
4
5
expenditure quintiles
bovine (mainly beef)
dried, smoked or salted
other fresh
other preserved
pork
poultry
sheep & goat

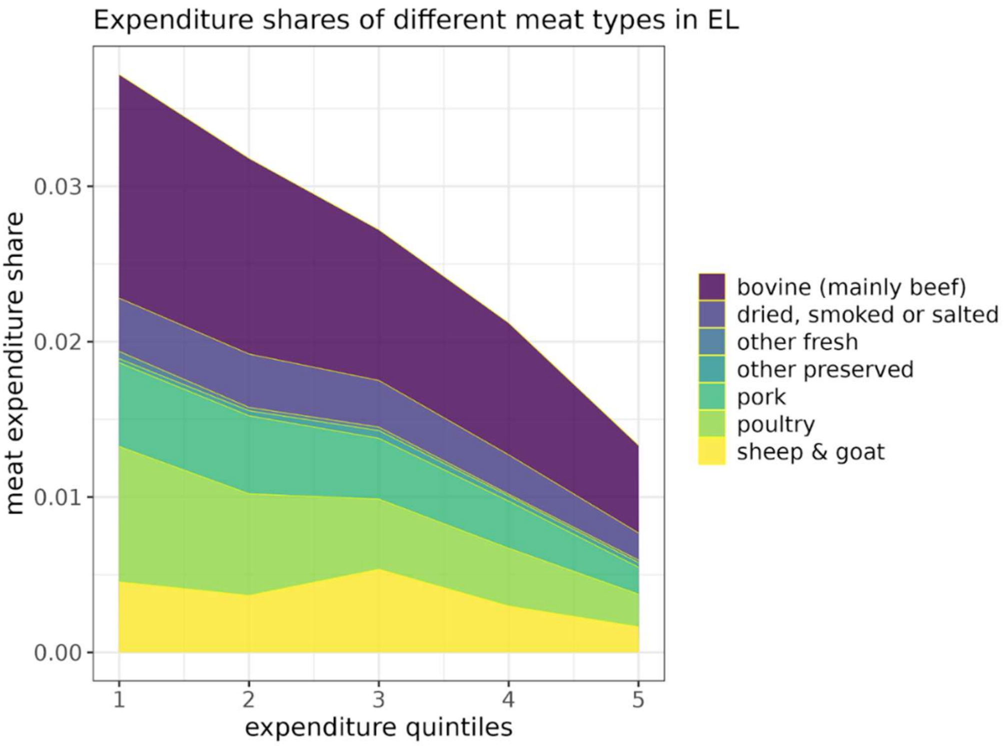
Expenditure shares of different meat types in EL
meat expenditure share
0.03
0.02
0.01
0.00
1
2
3
4
5
expenditure quintiles
bovine (mainly beef)
dried, smoked or salted
other fresh
other preserved
pork
poultry
sheep & goat

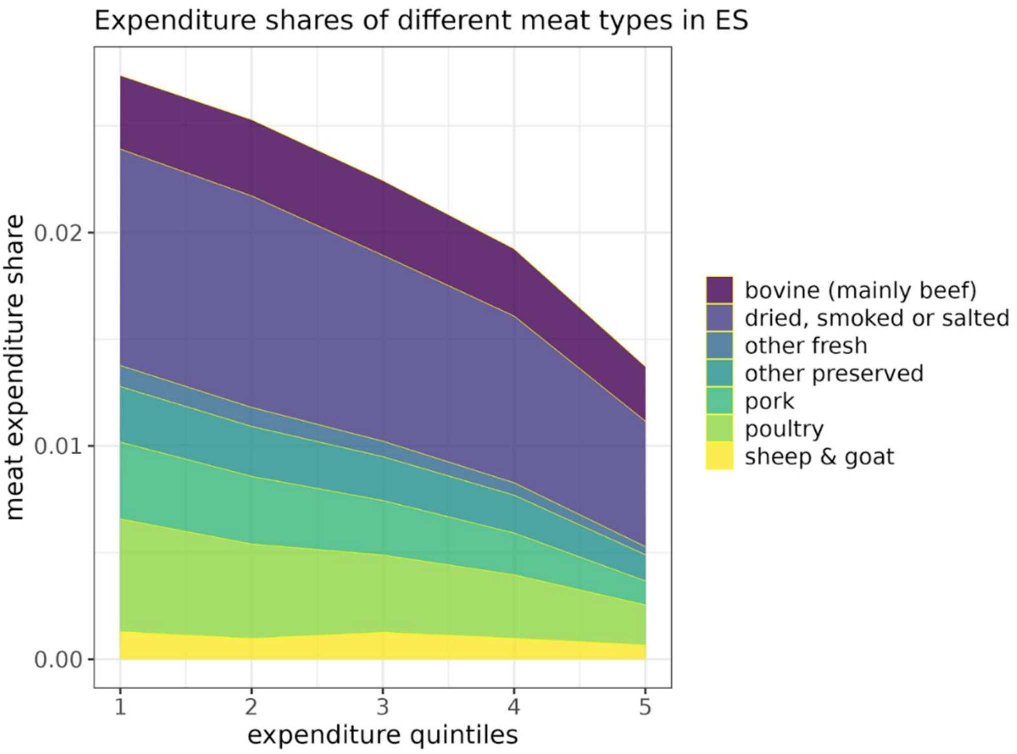
Expenditure shares of different meat types in ES
meat expenditure share
0.02
0.01
0.00
1
2
3
4
5
expenditure quintiles
bovine (mainly beef)
dried, smoked or salted
other fresh
other preserved
pork
poultry
sheep & goat

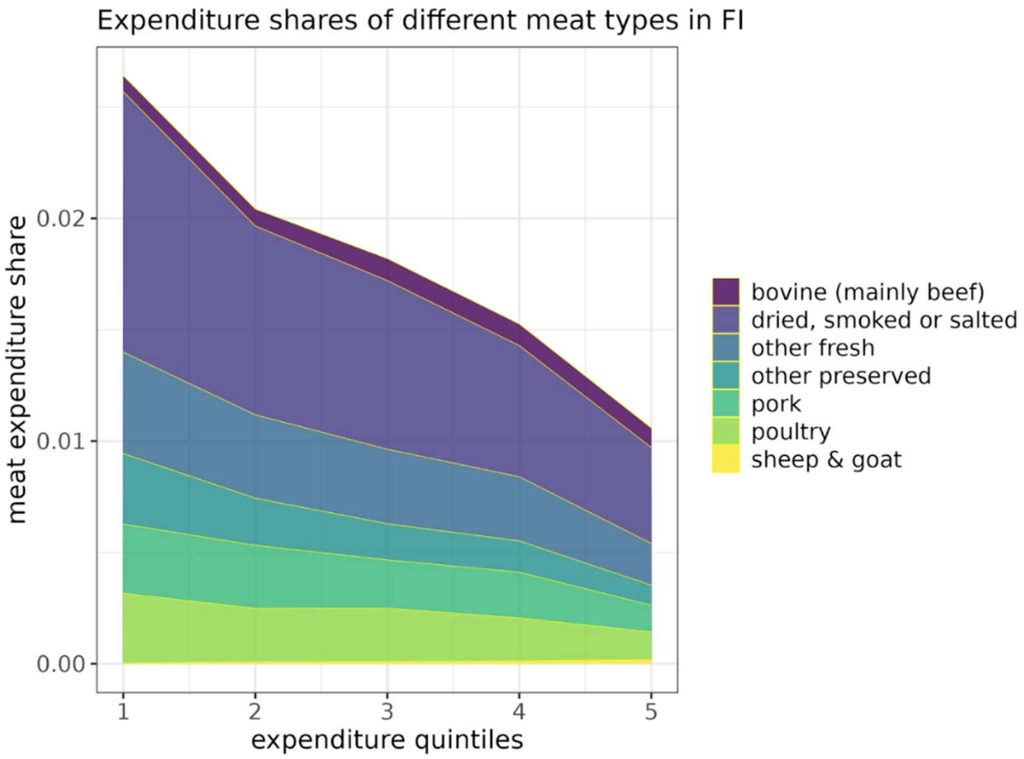
Expenditure shares of different meat types in FI
meat expenditure share
0.02
0.01
0.00
1
2
3
4
5
expenditure quintiles
bovine (mainly beef)
dried, smoked or salted
other fresh
other preserved
pork
poultry
sheep & goat

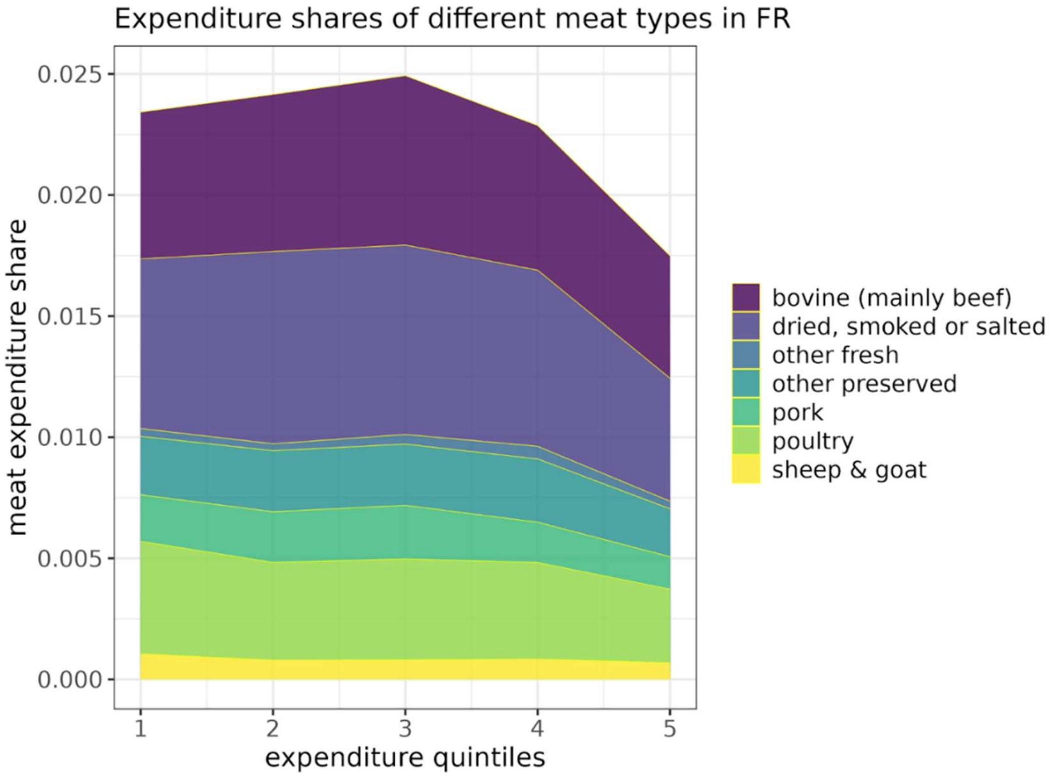
Expenditure shares of different meat types in FR
meat expenditure share
0.025
0.020
0.015
0.010
0.005
0.000
1
2
3
4
5
expenditure quintiles
bovine (mainly beef)
dried, smoked or salted
other fresh
other preserved
pork
poultry
sheep & goat

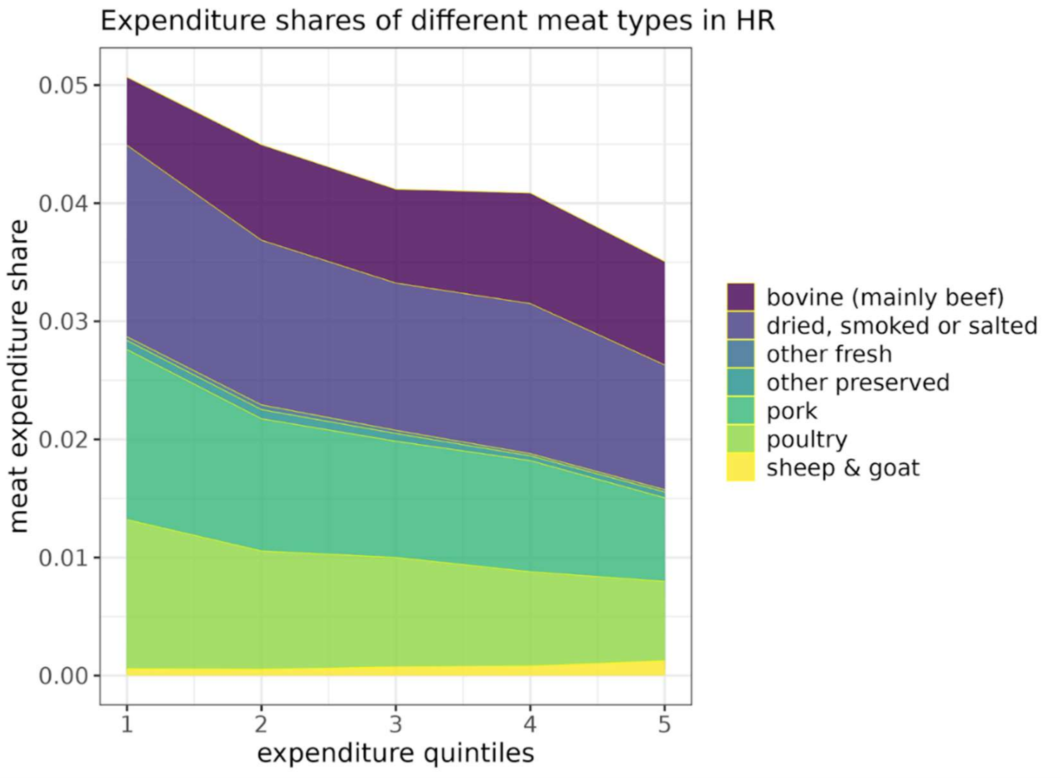
Expenditure shares of different meat types in HR
meat expenditure share
0.00
0.01
0.02
0.03
0.04
0.05
1
2
3
4
5
expenditure quintiles
bovine (mainly beef)
dried, smoked or salted
other fresh
other preserved
pork
poultry
sheep & goat

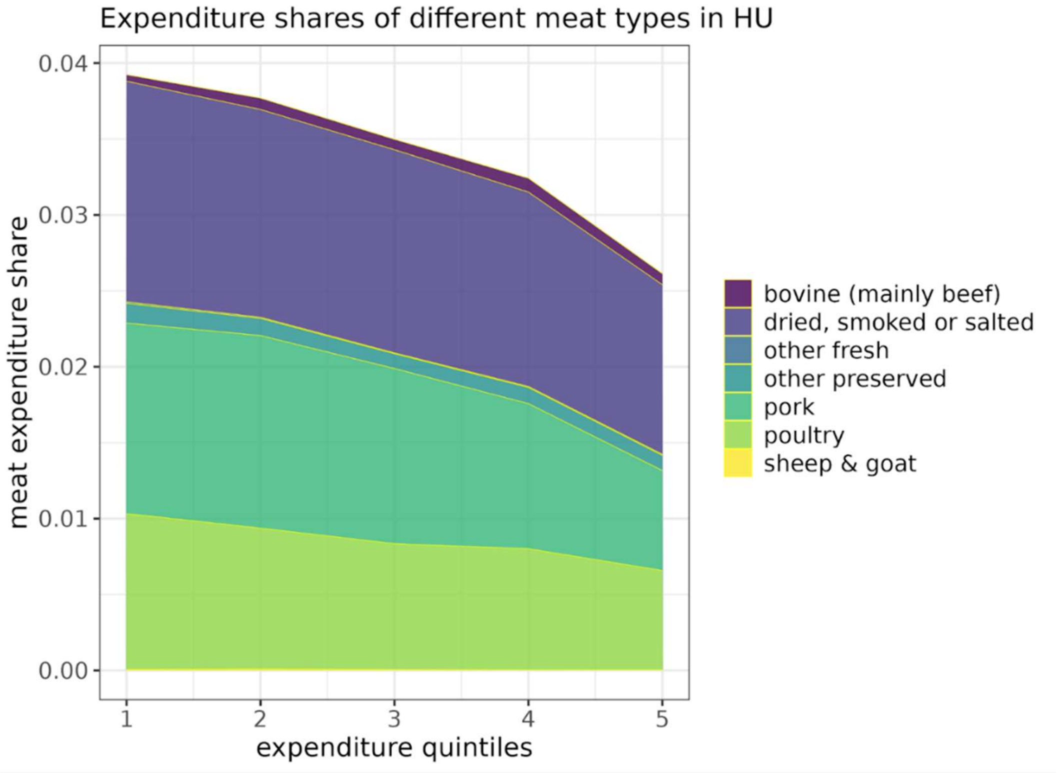
Expenditure shares of different meat types in HU
meat expenditure share
0.00
0.01
0.02
0.03
0.04
1
2
3
4
5
expenditure quintiles
bovine (mainly beef)
dried, smoked or salted
other fresh
other preserved
pork
poultry
sheep & goat

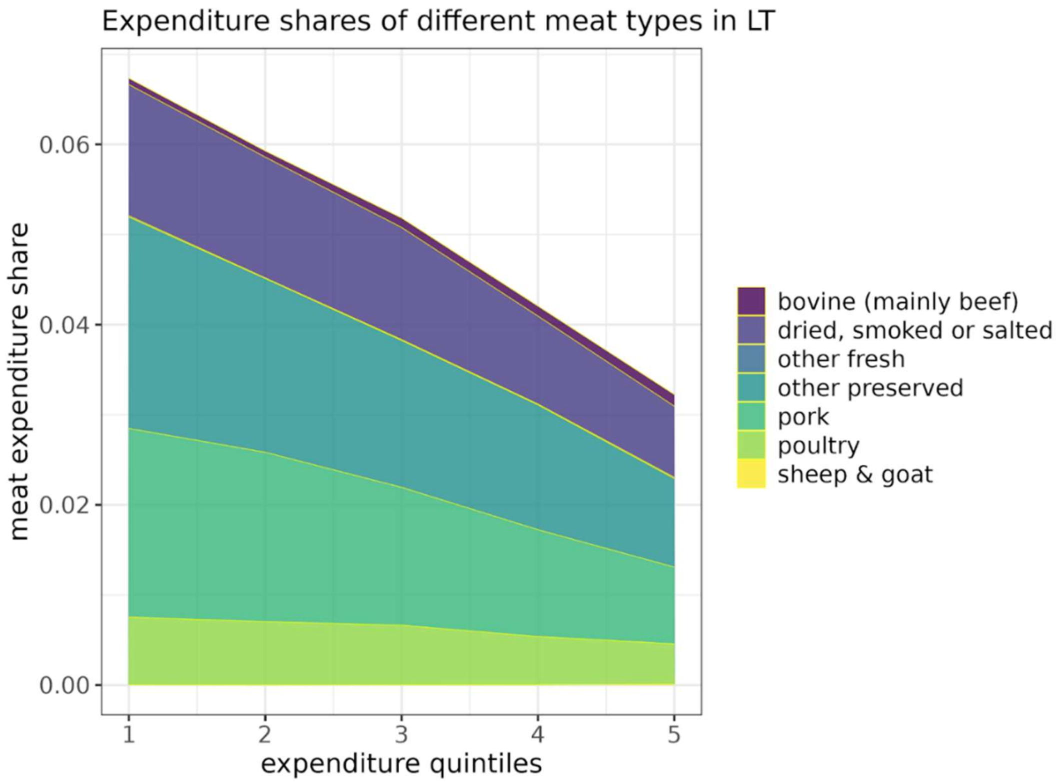
Expenditure shares of different meat types in LT
meat expenditure share
0.06
0.04
0.02
0.00
1
2
3
4
5
expenditure quintiles
bovine (mainly beef)
dried, smoked or salted
other fresh
other preserved
pork
poultry
sheep & goat

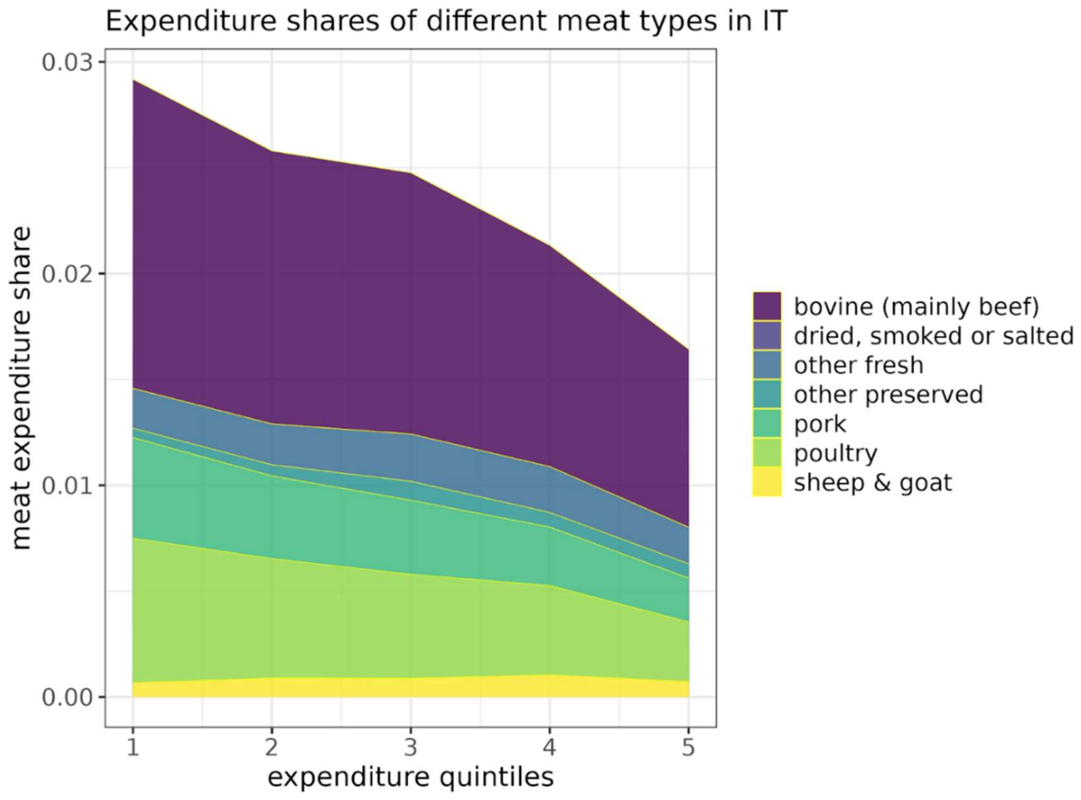
Expenditure shares of different meat types in IT
meat expenditure share
0.03
0.02
0.01
0.00
1
2
3
4
5
expenditure quintiles
bovine (mainly beef)
dried, smoked or salted
other fresh
other preserved
pork
poultry
sheep & goat

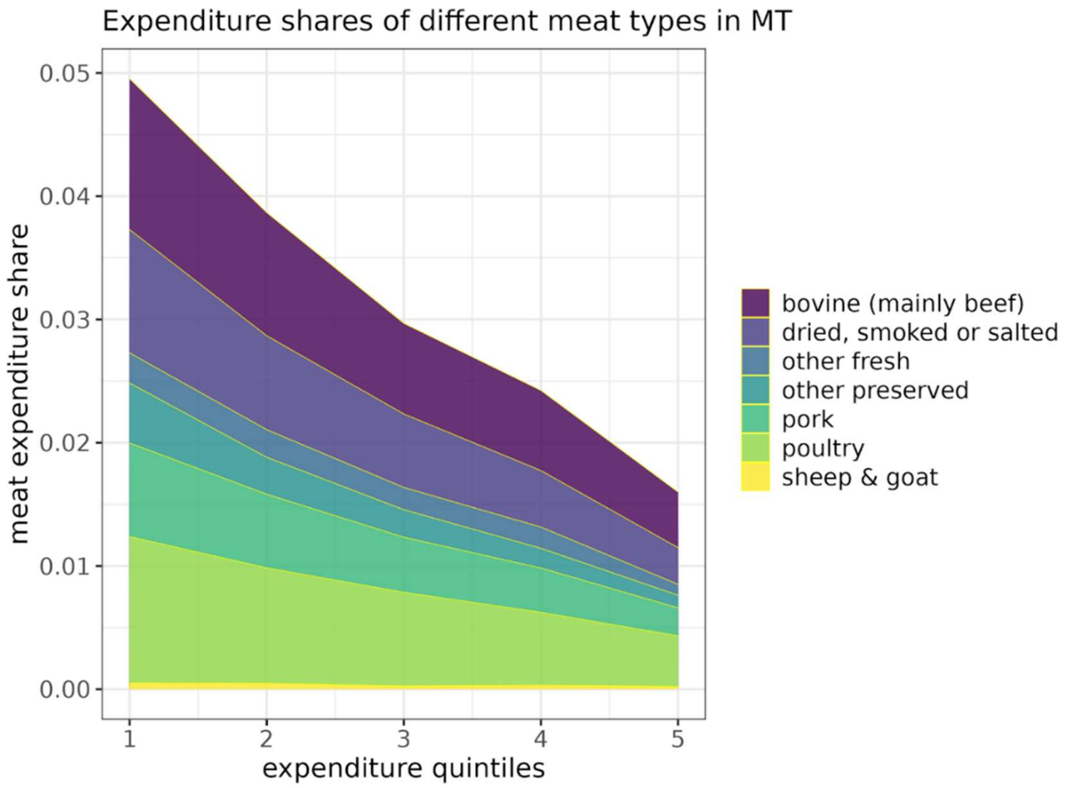
Expenditure shares of different meat types in MT
meat expenditure share
0.05
0.04
0.03
0.02
0.01
0.00
1
2
3
4
5
expenditure quintiles
bovine (mainly beef)
dried, smoked or salted
other fresh
other preserved
pork
poultry
sheep & goat

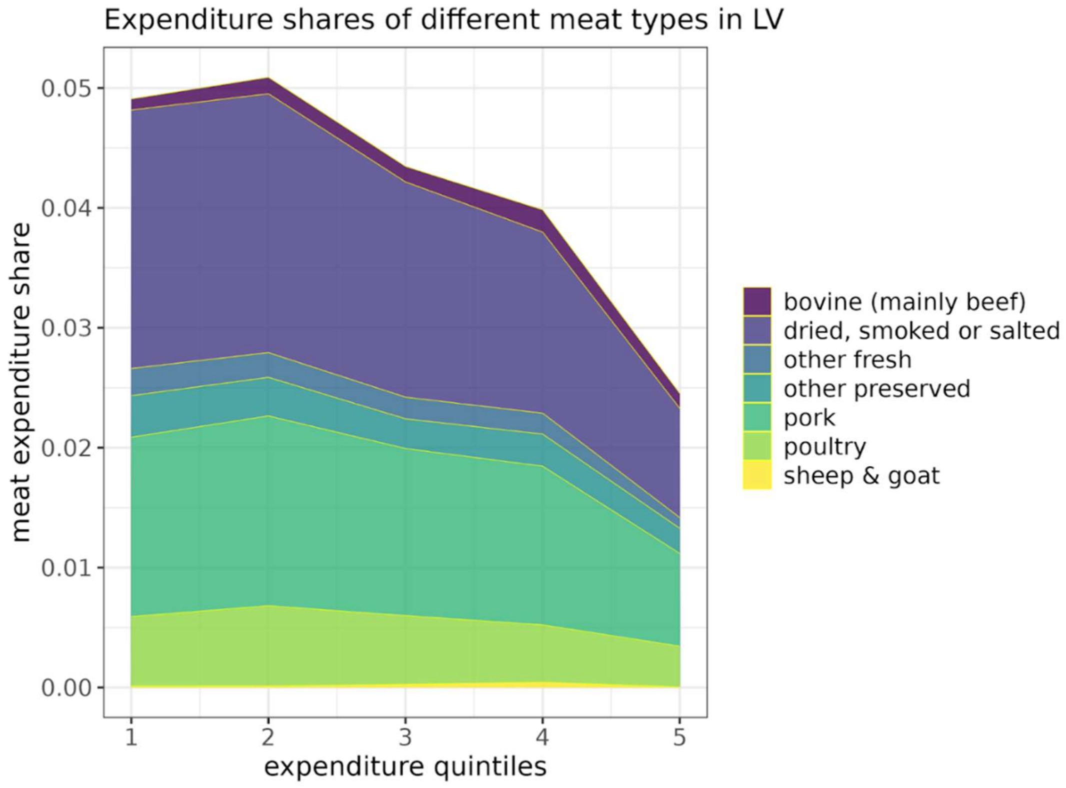
Expenditure shares of different meat types in LV
meat expenditure share
0.05
0.04
0.03
0.02
0.01
0.00
1
2
3
4
5
expenditure quintiles
bovine (mainly beef)
dried, smoked or salted
other fresh
other preserved
pork
poultry
sheep & goat

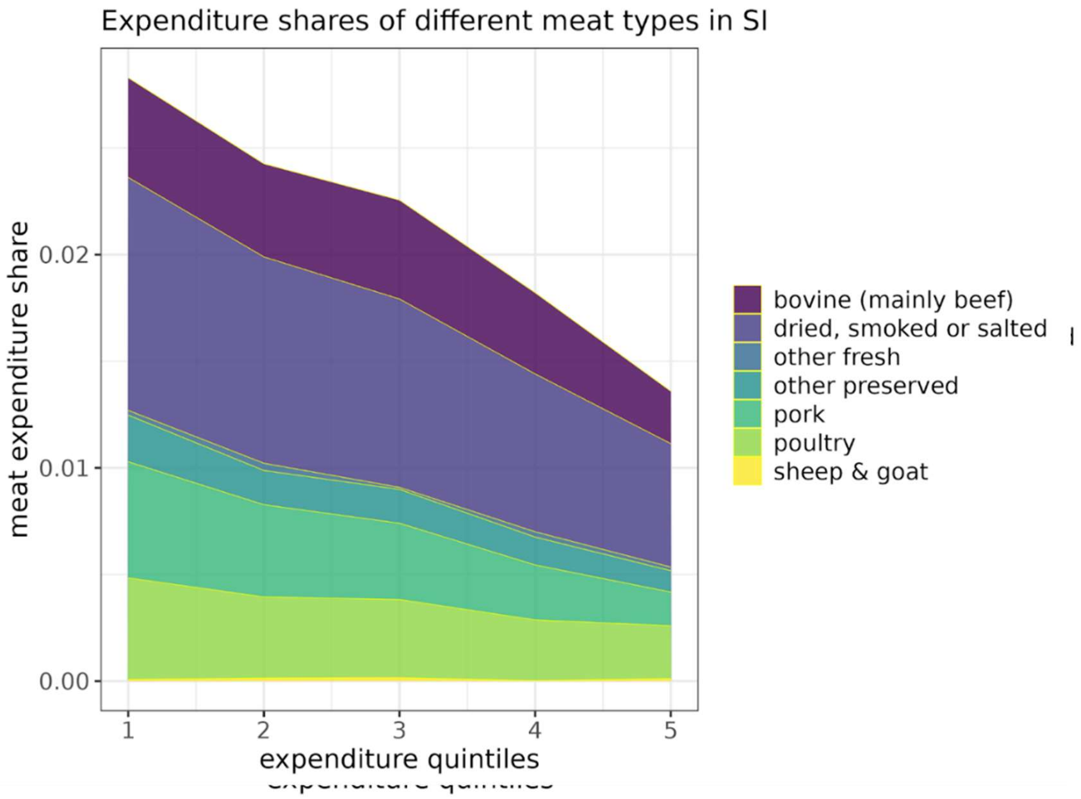
Expenditure shares of different meat types in SI
meat expenditure share
0.00
0.01
0.02
1
2
3
4
5
expenditure quintiles
bovine (mainly beef)
dried, smoked or salted
other fresh
other preserved
pork
poultry
sheep & goat

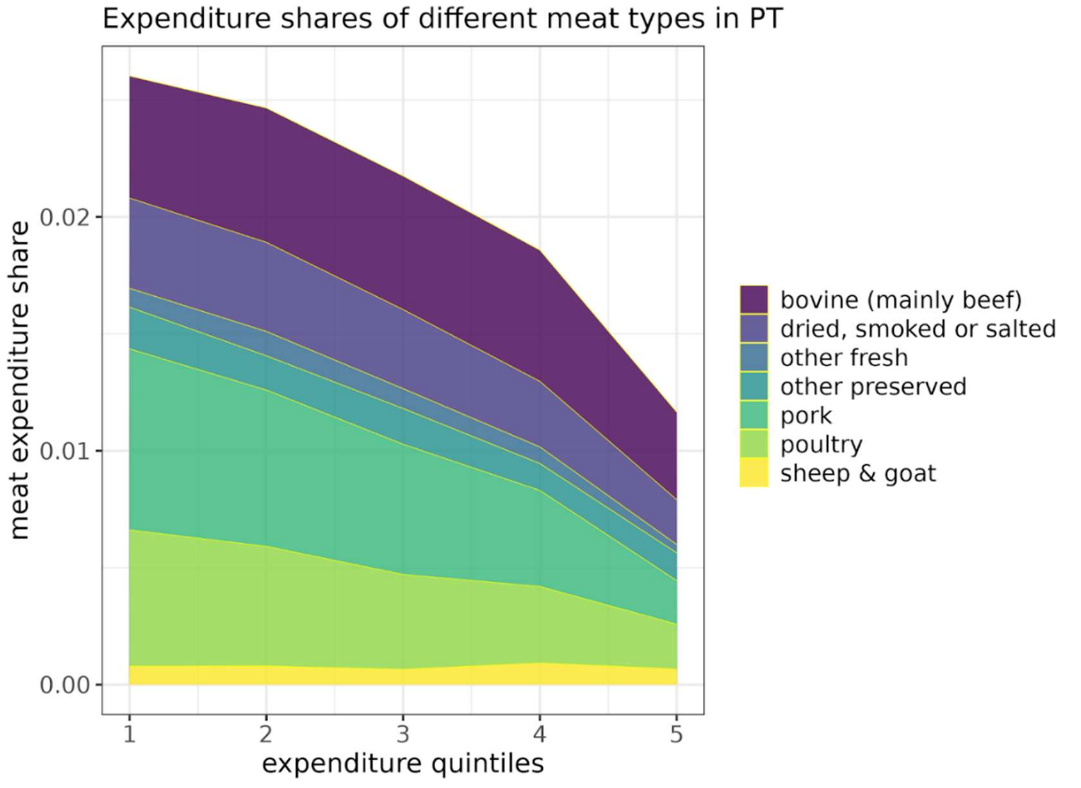
Expenditure shares of different meat types in PT
meat expenditure share
0.00
0.01
0.02
1
2
3
4
5
expenditure quintiles
bovine (mainly beef)
dried, smoked or salted
other fresh
other preserved
pork
poultry
sheep & goat

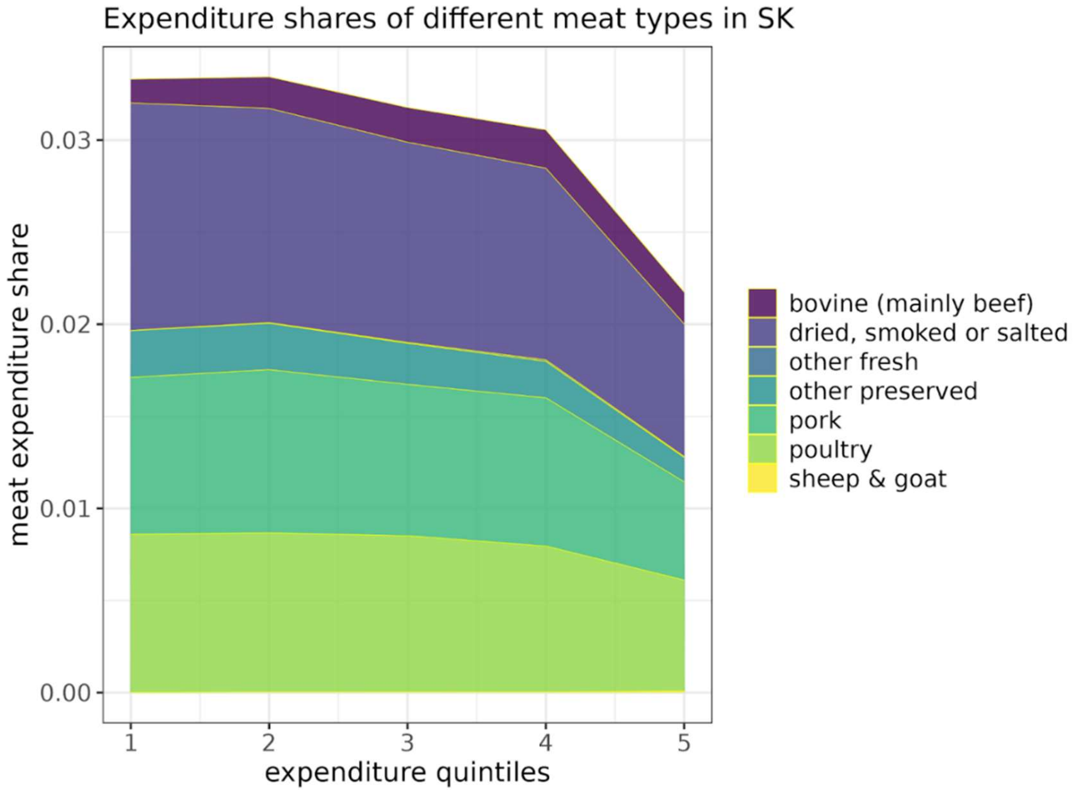
Expenditure shares of different meat types in SK
meat expenditure share
0.00
0.01
0.02
0.03
expenditure quintiles
1
2
3
4
5
bovine (mainly beef)
dried, smoked or salted
other fresh
other preserved
pork
poultry
sheep & goat

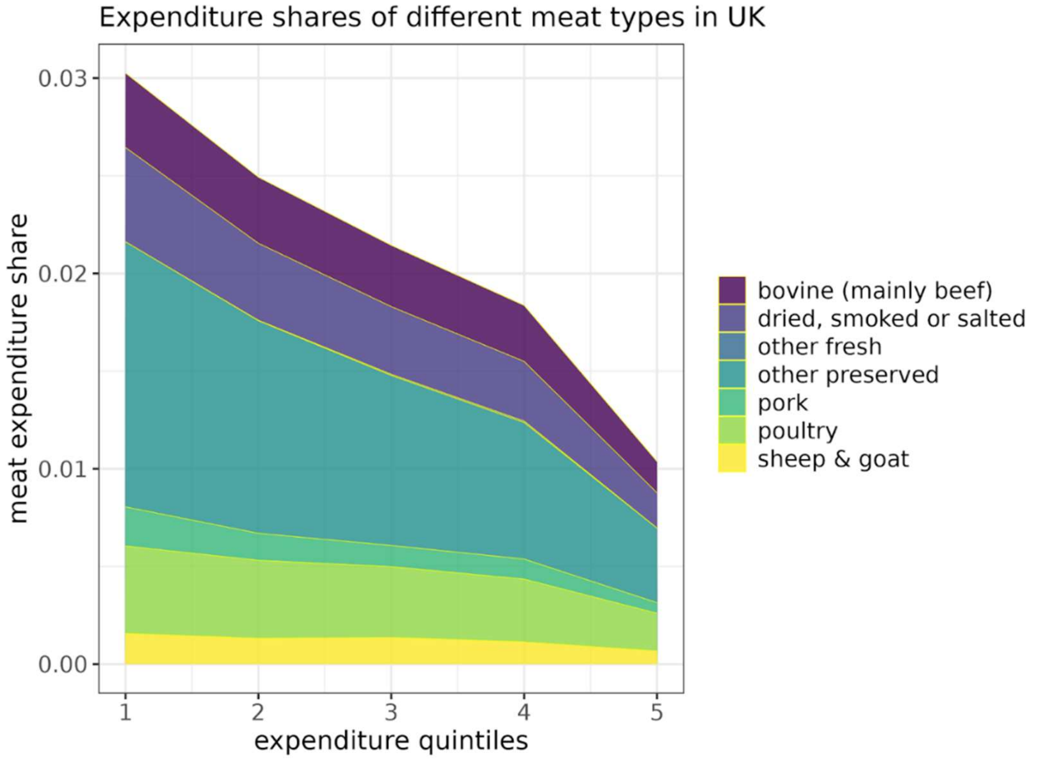
Expenditure shares of different meat types in UK
meat expenditure share
0.00
0.01
0.02
0.03
expenditure quintiles
1
2
3
4
5
bovine (mainly beef)
dried, smoked or salted
other fresh
other preserved
pork
poultry
sheep & goat

9. References

Bonnet, C., Bouamra-Mechemache, Z., & Corre, T. (2018). An environmental tax towards more sustainable food: empirical evidence of the consumption of animal products in France. *Ecological Economics*, *147*, 48–61.

EU Commission (2020). *VAT rates applied in the member states of the European Union. Situation as of 1 January 2020*. Available at: https://taxation-customs.ec.europa.eu/system/files/2020-10/vat_rates_en.pdf

Gallet, C. A. (2010). Meat meets meta: a quantitative review of the price elasticity of meat. *American Journal of Agricultural Economics*, *92*(1), 258–272.
