# Supplementary material for: Meat taxes in Europe can be designed to avoid overburdening low-income consumers
Source: Nat Food. 2023 Oct 2;4(10):894–901. doi: 10.1038/s43016-023-00849-z (PMC10589082; doi:10.1038/s43016-023-00849-z)
Supplement: Supplementary file 2 — Reporting Summary [file 43016_2023_849_MOESM2_ESM.pdf]

nature portfolio

Corresponding author(s): David Klenert, Franziska Funke, Mattia Cai

Last updated by author(s): Sep 1, 2023

Reporting Summary

Nature Portfolio wishes to improve the reproducibility of the work that we publish. This form provides structure for consistency and transparency in reporting. For further information on Nature Portfolio policies, see our Editorial Policies and the Editorial Policy Checklist.

Statistics

For all statistical analyses, confirm that the following items are present in the figure legend, table legend, main text, or Methods section.

n/a	Confirmed	
☒	☐	The exact sample size (*n*) for each experimental group/condition, given as a discrete number and unit of measurement
☒	☐	A statement on whether measurements were taken from distinct samples or whether the same sample was measured repeatedly
☒	☐	The statistical test(s) used AND whether they are one- or two-sided<br>*Only common tests should be described solely by name; describe more complex techniques in the Methods section.*
☒	☐	A description of all covariates tested
☒	☐	A description of any assumptions or corrections, such as tests of normality and adjustment for multiple comparisons
☐	☒	A full description of the statistical parameters including central tendency (e.g. means) or other basic estimates (e.g. regression coefficient) AND variation (e.g. standard deviation) or associated estimates of uncertainty (e.g. confidence intervals)
☒	☐	For null hypothesis testing, the test statistic (e.g. *F*, *t*, *r*) with confidence intervals, effect sizes, degrees of freedom and *P* value noted<br>*Give P values as exact values whenever suitable.*
☒	☐	For Bayesian analysis, information on the choice of priors and Markov chain Monte Carlo settings
☒	☐	For hierarchical and complex designs, identification of the appropriate level for tests and full reporting of outcomes
☒	☐	Estimates of effect sizes (e.g. Cohen's *d*, Pearson's *r*), indicating how they were calculated

Our web collection on statistics for biologists contains articles on many of the points above.

Software and code

Policy information about availability of computer code

Data collection	We use the EU Household Budget Survey (EU HBS) so no software was necessary for data collection.
Data analysis	We use R (mostly version 3.6.0.) to clean and aggregate the data and to do the microsimulations. We use Stata (release 17) to calculate the 95% confidence intervals. The code is publicly available under the following DOI: 10.5281/zenodo.8308085

For manuscripts utilizing custom algorithms or software that are central to the research but not yet described in published literature, software must be made available to editors and reviewers. We strongly encourage code deposition in a community repository (e.g. GitHub). See the Nature Portfolio guidelines for submitting code & software for further information.

Data

Policy information about availability of data

All manuscripts must include a data availability statement. This statement should provide the following information, where applicable:

- Accession codes, unique identifiers, or web links for publicly available datasets
- A description of any restrictions on data availability
- For clinical datasets or third party data, please ensure that the statement adheres to our policy

Data availability statement: Our main source of data is Eurostat’s Household Budget Survey (https://ec.europa.eu/eurostat/web/microdata/household-budget-survey). Researchers can request access to these data under specific conditions laid out in this document (https://ec.europa.eu/eurostat/documents/203647/771732/How_to_apply_for_microdata_access.pdf). Additional data on population are

available through Eurostat's Population and Democracy database (https://ec.europa.eu/eurostat/web/population-demography/demography-population-stock-balance/database). Data on VAT rates in the EU are reprinted in Supplementary Table 5.

Human research participants

Policy information about studies involving human research participants and Sex and Gender in Research.

Reporting on sex and gender	This information is not contained in the European Household Budget Survey as data is collected on the household level
Population characteristics	See above.
Recruitment	See the EU HBS manual available at: https://ec.europa.eu/eurostat/web/microdata/household-budget-survey<br><br>Most of the participating countries draw a sample of households in a way that the probability of a household being selected is known (technically known as a probability design). In this way, the results can be reliably projected from the sample to the household reference population with known levels of precision, i.e. standard errors and confidence intervals for survey estimates can be constructed.
Ethics oversight	The analysis is based on existing household consumption data (EU HBS). When requesting access to these data, our research proposal was approved by Eurostat.

Note that full information on the approval of the study protocol must also be provided in the manuscript.

Field-specific reporting

Please select the one below that is the best fit for your research. If you are not sure, read the appropriate sections before making your selection.

☐ Life sciences ☒ Behavioural & social sciences ☐ Ecological, evolutionary & environmental sciences

For a reference copy of the document with all sections, see nature.com/documents/nr-reporting-summary-flat.pdf

Behavioural & social sciences study design

All studies must disclose on these points even when the disclosure is negative.

Study description	In the study we use (quantitative) micro-economic data on household expenditure and consumption patterns from the European Household Budget Survey (EU HBS) to analyse the distributional effects of different meat tax designs using microsimulations.
Research sample	The EU HBS is an existing dataset that provides expenditure data on households in the EU27+UK with the exception of households in Austria, Germany and the Netherlands, which do not provide sufficiently detailed data for our analysis. Source: https://ec.europa.eu/eurostat/web/microdata/household-budget-survey<br><br>Most of the participating countries draw a sample of households in a way that the probability of a household being selected is known (technically known as a probability design). In this way, the results can be reliably projected from the sample to the household reference population with known levels of precision, i.e. standard errors and confidence intervals for survey estimates can be constructed. For more details see the HBS 2010/2015 Wave EU Quality report http://ec.europa.eu/eurostat/web/household-budget-surveys/publications<br><br>The EU HBS is that it is the most comprehensive source for household consumption expenditures in the EU and is used widely in the literature for analysing distributional questions (as we do in this article).
Sampling strategy	The sampling frames adopted for the HBS in Europe range from population registers, which can provide up-to-date lists of households or individuals with characteristics useful for stratification and efficient selection of the sample, to Censuses or list of addresses. Sampling design varies from two stage stratified random samples to simple random samples; CZ & DE uses quota sampling.<br>Details regarding the sampling characteristics, individual countries' sample sizes and weights adopted by the MSs are available in the HBS 2010/2015 Wave EU Quality report http://ec.europa.eu/eurostat/web/household-budget-surveys/publications
Data collection	HBS data collection involves, most of the time, a combination of one or more interviews, and diaries or logs maintained by households and/or individuals, generally on a daily basis. The period for which a diary is maintained is called the recording period. The duration and distribution of this time period is the most important determinant of the structure of the survey. The other time period that characterizes the HBS is the survey period: it is the period of time for which the household consumption expenditure is recorded. The survey periods may vary from one year to multiple years (two or three years) depending of the nature of survey. To facilitate the creation of Aggregates at an EU level, each countries data transmission is mapped to an agreed Reference Year (2010).

The researcher collecting the data was not aware of our research question, as we use an existing dataset.

Timing	See "Data Collection", above.
Data exclusions	No valid data were excluded. But in the course of the cleaning of the data, we filtered out negative expenditure values and clear accounting errors, in particular per capita consumption levels of meat above 500 kg per year.
Non-participation	According to the Quality Report for the 2010 wave of the EU HBS, the mean response rate at EU level lies around 60%. There are however important variations between the countries: from 5.6% in Belgium to 87.6% in Romania. (The low figure for BE is explained by the fact that it is calculated taking in to account the total population of households initially contacted, to participate in the HBS.).
Randomization	This is not relevant for our study, because we do a microsimulation of one large sample, before and after a policy intervention. There are no experimental groups, only the sample before and after the intervention.

Reporting for specific materials, systems and methods

We require information from authors about some types of materials, experimental systems and methods used in many studies. Here, indicate whether each material, system or method listed is relevant to your study. If you are not sure if a list item applies to your research, read the appropriate section before selecting a response.

Materials & experimental systems

n/a	Involved in the study
☒	☐ Antibodies
☒	☐ Eukaryotic cell lines
☒	☐ Palaeontology and archaeology
☒	☐ Animals and other organisms
☒	☐ Clinical data
☒	☐ Dual use research of concern

Methods

n/a	Involved in the study
☒	☐ ChIP-seq
☒	☐ Flow cytometry
☒	☐ MRI-based neuroimaging
